# Zn(II) binding causes interdomain changes in the structure and flexibility of the human prion protein

**DOI:** 10.1038/s41598-021-00495-0

**Published:** 2021-11-04

**Authors:** Maciej Gielnik, Michał Taube, Lilia Zhukova, Igor Zhukov, Sebastian K. T. S. Wärmländer, Željko Svedružić, Wojciech M. Kwiatek, Astrid Gräslund, Maciej Kozak

**Affiliations:** 1 Department of Macromolecular Physics, Faculty of Physics, Adam Mickiewicz University, 61-614 Poznań, Poland; 2 Institute of Biochemistry and Biophysics, Polish Academy of Sciences, 02-106 Warszawa, Poland; 3 Department of Biochemistry and Biophysics, Stockholm University, 10691 Stockholm, Sweden; 4 Department of Biotechnology, University of Rijeka, 51000 Rijeka, Croatia; 5 Institute of Nuclear Physics Polish Academy of Sciences, 31-342 Kraków, Poland; 6 National Synchrotron Radiation Centre SOLARIS, Jagiellonian University, 30-392 Kraków, Poland

**Keywords:** Intrinsically disordered proteins, Prions

## Abstract

The cellular prion protein (PrP^C^) is a mainly α-helical 208-residue protein located in the pre- and postsynaptic membranes. For unknown reasons, PrP^C^ can undergo a structural transition into a toxic, β-sheet rich scrapie isoform (PrP^Sc^) that is responsible for transmissible spongiform encephalopathies (TSEs). Metal ions seem to play an important role in the structural conversion. PrP^C^ binds Zn(II) ions and may be involved in metal ion transport and zinc homeostasis. Here, we use multiple biophysical techniques including optical and NMR spectroscopy, molecular dynamics simulations, and small angle X-ray scattering to characterize interactions between human PrP^C^ and Zn(II) ions. Binding of a single Zn(II) ion to the PrP^C^ N-terminal domain via four His residues from the octarepeat region induces a structural transition in the C-terminal α-helices 2 and 3, promotes interaction between the N-terminal and C-terminal domains, reduces the folded protein size, and modifies the internal structural dynamics. As our results suggest that PrP^C^ can bind Zn(II) under physiological conditions, these effects could be important for the physiological function of PrP^C^.

## Introduction

Misfolding and aggregation of the 208-residue prion protein (PrP) is the molecular event underlying the progressive and fatal neurodegenerative diseases collectively known as transmissible spongiform encephalopathies (TSEs)^1^, or prion diseases. PrP is an outer membrane glycoprotein encoded by the PRNP gene, highly conserved within mammals^2^, and expressed at high levels in the brain—especially in the striatum, hippocampus and cortex^3^. Single-nucleotide mutations or sequence expansions within the PRNP gene are the cause of genetic prion diseases such as familial Creutzfeldt-Jakob disease (fCJD), fatal familial insomnia (FFI), and Gerstmann-Sträussler-Scheinker (GSS) syndrome. Post-translational modifications of PrP produce the mature cellular form known as PrP^C^^4–6^, which can undergo a structural rearrangement into the aggregated, β-sheet-rich, and pathological (scrapie) isoform denoted as PrP^Sc^. This form acts as a template for PrP^C^ to refold into toxic conformations^1,7^. The infectious prion diseases arise from contact with pathogenic PrP^Sc^ via events such as organ transplantation from people with CJD (iatrogenic CJD), consumption of beef contaminated with bovine spongiform encephalopathy (variant CJD)^1,7^, or human cannibalistic rituals (Kuru)^8^. The most common prion disease in humans is however sporadic CJD, but its origins are unknown. Despite intense research, no drugs have so far been devised that can cure prion diseases^9–11^.

**Figure 1:**
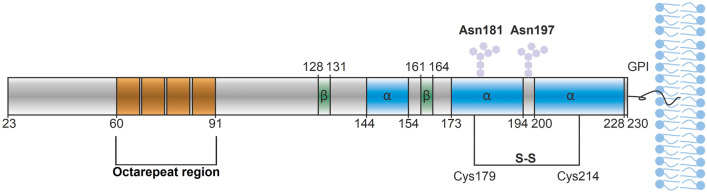
Schematic representation of the mature human PrP^C^ protein. PrP^C^ consists of an N-terminal intrinsically disordered domain and a C-terminal structured domain. The octarepeat (OR) region is marked in orange, α-helices are marked in blue, β-sheets are marked in green, hexagons represent the glycosylation sites, S–S is a disulfide bridge and GPI represents the glycosylphosphatidylinositol anchor.

The human PrP protein is expressed as a 253-residue long precursor polypeptide chain. The post-translational modifications include: removal of the N-terminal 22-residue signal sequence, removal of 23 C-terminal residues, formation of one disulfide bridge (Cys179-Cys214), glycosylation of two asparagine residues (Asn181, Asn197), as well as binding of a glycosylphosphatidylinositol (GPI) anchor^4–6^ (Fig. 1). The N-terminal domain of PrP^C^ is intrinsically disordered^12^, although it contains four octapeptide repeats with β-turn or polyproline II secondary structure^13–15^. The C-terminal domain, whose 3D-fold is well conserved within mammals, consists of three α-helices and two antiparallel β-sheets^12,16–18^. The first α-helix is formed by residues 144–154 and is flanked by two short β-strands, i.e. residues 128–131 and 161–164. Helices α2 and α3 consist of residues 173–194 and 200–228, and are interconnected by a disulfide bond between Cys179 and Cys214^12,18,19^. In vitro studies suggest that conversion of the mainly α-helical PrP^C^ into the toxic and β-sheet-rich PrP^Sc^ isoform requires misfolding or unfolding of PrP^C^ as an intermediate step^1,7^.

The biological function of PrP^C^ is not fully understood. The protein is involved in controlling synaptic transmission and neuronal plasticity. Studies on mouse models suggest that PrP^C^ may be crucial for the regulation of the circadian rhythm, and the development of the central nervous system^20,21^. Moreover, it has been proposed that PrP^C^ might have a significant role in the homeostasis of different metal ions^21,22^, as the brain distribution of metal ions correlates with the PrP^C^ expression level^23^. It is still unclear if zinc dyshomeostasis, or metal imbalances in general, are part of the pathology in prion diseases, as appears to be the case in other neurodegenerative protein aggregation diseases such as Alzheimer's ^24^.

Human PrP^C^ has been tested for binding of various divalent metal ions including Cu(II), Ni(II), Zn(II), and Mn(II)^25–30^. Zinc is the second-most abundant (after iron) metal in living organisms. It is a co-factor of many enzymes^31^, and plays an important role in cell signaling and proliferation^32^. Zn(II) ions are important neurotransmitters in the synaptic cleft where concentrations can reach up to 300 µM^33,34^. In PrP^C^ both Cu(II) and Zn(II) mainly binds to the octarepeat region (Fig. 1, orange)^25,29,35^. Upon binding of Zn(II) ions, the N-terminal domain forms a tertiary contact with the C-terminal domain via the octarepeats^36^. This interaction is disrupted in PrP^C^ mutants associated with TSE, suggesting that disruption of Zn(II)-mediated intramolecular interactions might cause TSE^36^. Even though the interaction between PrP^C^ and Zn(II) has been investigated for the last twenty years, the dissociation constant values reported in literature for the formed complex vary in almost three orders of magnitude, i.e. from ~ 0.5 μM to ~ 200 μM, even when measured in similar environments^25,37,38^.

Here, we used multiple biophysical techniques, including spectroscopic, scattering, and theoretical methods, to study Zn(II) binding to the full-length human PrP^C^ protein.

## Results

### CD spectroscopy reveals α-helix to β-sheet transition in PrP^C^ upon Zn(II) binding

**Figure 2:**
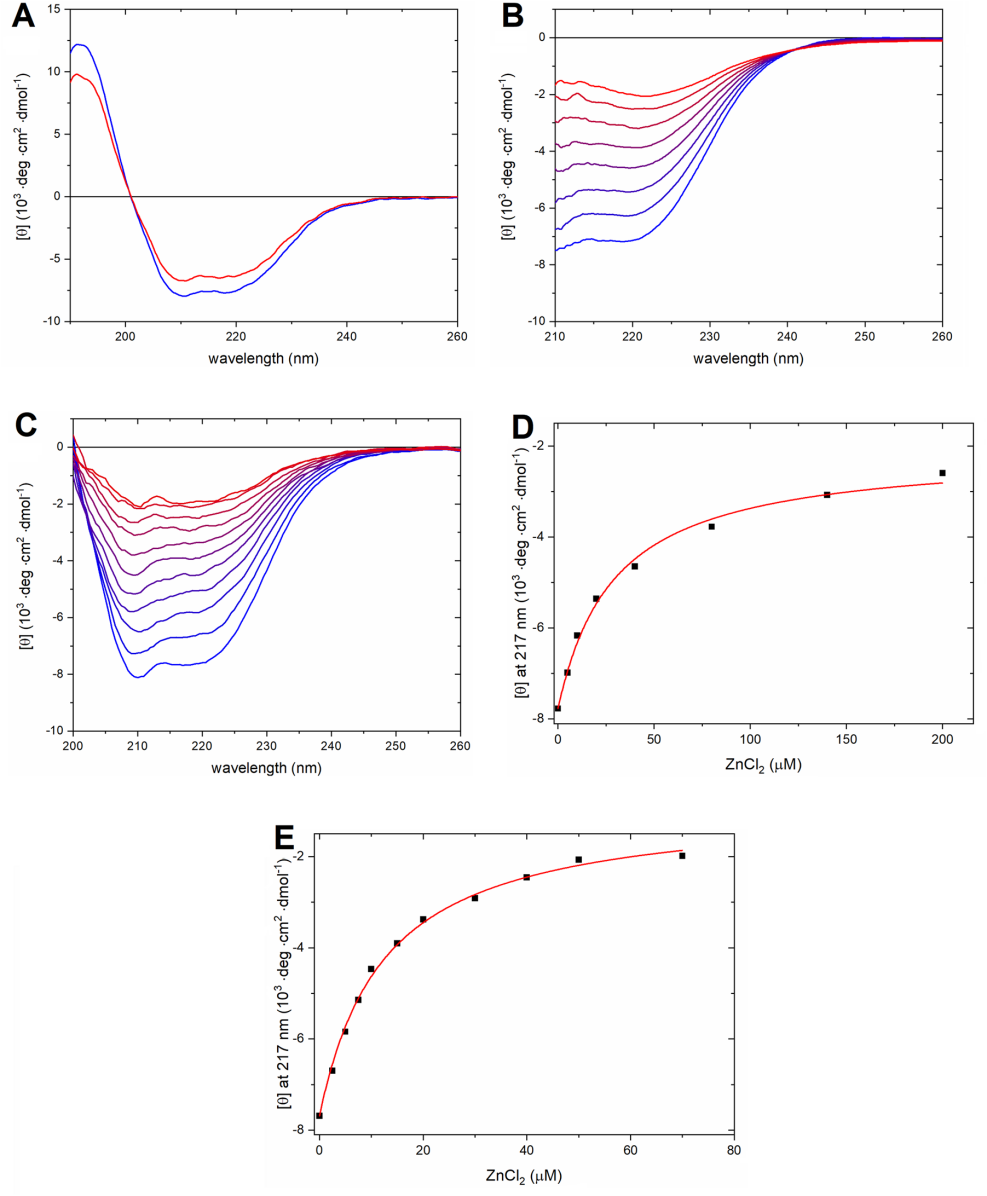
CD spectra of *hu*PrP^C^(23–231) titrated with ZnCl_2_. **(A)** 20 μM PrP^C^ (blue line) and 20 μM PrP^C^ with 400 μM ZnCl_2_ (red line) in 10 mM NEM buffer, pH 7.4. **(B)** Titration of 1 μM PrP^C^ with ZnCl_2_ in 10 mM NEM buffer, pH 7.4. The initial spectrum is in blue and the final one in red. **(C)** Titration of 0.5 μM PrP^C^ with ZnCl_2_ in 10 mM sodium phosphate buffer, pH 7.4. The initial spectrum is in blue and the final spectrum is in red. **(D)** CD signal intensity at 217 nm derived from the spectra in Fig. 2B, plotted as a function of added ZnCl_2_ and fitted to Eq. (1) (K_d_ = 28.8 ± 1.5 μM). **(E)** CD signal intensity at 217 nm derived from the spectra in (C), plotted as a function of added ZnCl_2_ and fitted to Eq. (1) (K_d_ = 12.1 ± 0.7 μM).

Circular dichroism (CD) spectroscopy was used to monitor changes in protein secondary structure induced by Zn(II) ions. The CD spectrum of the pure protein in N-ethyl morpholine (﻿NEM) buffer corresponds to a typical α-helix, with characteristic minima at 208 and 222 nm and a maximum at 193 nm (Fig. 2A). Addition of twenty molar equivalents of Zn(II) to PrP^C^ resulted in a general decrease in CD intensity over the whole wavelength range (Fig. 2A). The observed change corresponds to a decrease in the content of regular α-helices (helix 1, Table S1), and an increase in the content of distorted α-helices (helix 2, Table S1). Addition of Zn(II) also increased the content of antiparallel β-sheets and β-turns, and reduced the content of parallel β-sheets (Table S1). The changes in PrP^C^ secondary structure upon addition of Zn(II) were clearly visible in the far UV region; we therefore proceeded with careful Zn(II) titrations in this region.

ZnCl_2_ was titrated to PrP^C^, both in 10 mM NEM, pH 7.4 (Fig. 2B), and in 10 mM sodium phosphate buffer, pH 7.4 (Fig. 2C). The measurements in NEM buffer displayed an isodichroic point at ~ 242 nm (Fig. 2B). In phosphate buffer no isodichroic point was visible (Fig. 2C). The presence of this isodichroic point shows that the Zn(II)-induced general loss of CD signal intensity is caused by a two-phase structural transition, and not by a lowered protein concentration related to e.g. possible Zn-related aggregation and precipitation of the protein. Figure 2D,E show plots of the CD intensity at 217 nm vs Zn(II) concentration, derived from the spectra in Fig. 2B,C. Fitting binding Eq. (1) to the Zn(II) titration data in NEM buffer (Fig. 2B) produced a dissociation constant (K_d_) of 28.8 ± 1.5 μM (Fig. 2D), while the titration data in sodium phosphate buffer (Fig. 2C) suggested stronger binding of Zn(II) to PrP^C^. Fitting to Eq. (1) yielded the dissociation constant K_d_ = 12.1 ± 0.7 μM (Fig. 2E). Clearly, the binding behavior of the Zn(II) ions is to some extent influenced by the environmental conditions, such as buffer composition. Because possible binding of Zn(II) ions to the buffer is not taken into account, the calculated K_d_ values should be considered to be apparent.

### Fluorescence spectroscopy shows that tryptophan residues are in close proximity to the PrP^C^-Zn(II) binding site

**Figure 3:**
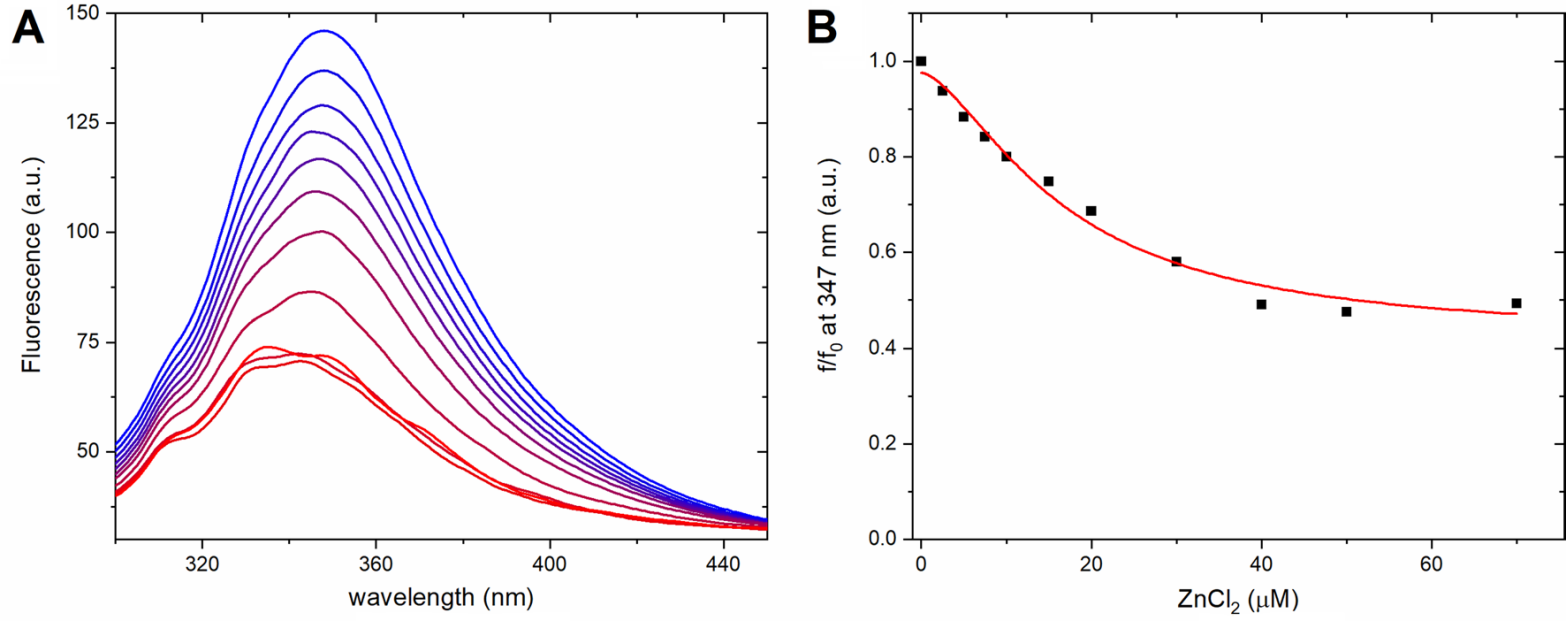
**(A)** Fluorescence spectra (excitation wavelength = 280 nm) of 0.5 μM *hu*PrP^C^ protein titrated with 0 to 70 µM ZnCl_2_, see **(B)**, in 10 mM sodium phosphate buffer, pH 7.4 at 25 °C. The initial spectrum is in blue and the final in red. **(B)** Changes in the relative fluorescence intensity at 347 nm, fitted to Eq. (2) (K_d_ = 16.8 ± 0.9 μM).

The PrP^C^ protein has seven tryptophan residues located in the N-terminal unstructured domain. Four of these residues, i.e. Trp65, Trp73, Trp81 and Trp89, are located in the octarepeat region where they appear to be indirectly involved in copper binding^35^. Addition of Cu(II) ions quench the tryptophan fluorescence signal^25^. Here, a similar effect is observed upon addition of Zn(II) ions to 0.5 μM PrP^C^ protein in 10 mM sodium phosphate buffer, pH 7.4 (Fig. 3A). The fluorescence spectrum of *apo*-PrP^C^ showed a single maximum at ~ 347 nm, indicating full exposure of the tryptophan residues to the solvent^39^.

The titration with ZnCl_2_ solution resulted in a reduced fluorescence intensity, a slight blue shift of the maximum to 343 nm, and revealed the presence of two additional peaks at 333 nm and 313 nm (Fig. 3A). The blue shift of tryptophan fluorescence to ~ 342 nm corresponds to a change in tryptophan environment and exposure to the bound water molecules, while the two new peaks at 333 nm and 313 nm may be attributed to buried and structured tryptophan residues, respectively^39^. In the last fluorescence spectrum of the titration, corresponding to a Zn(II) concentration of 70 µM, the maximum at 343 nm is still visible. Plotting the fluorescence intensity at 347 nm versus ZnCl_2_ concentration and fitting the data to Eq. (2) suggested binding of Zn(II) ions to the PrP^C^ protein (Fig. 3B) with an apparent dissociation constant K_d_ = 16.8 ± 0.9 μM.

### NMR spectroscopy shows Zn(II) binding promotes interactions between the PrP^C^ N- and C-terminal domains

**Figure 4:**
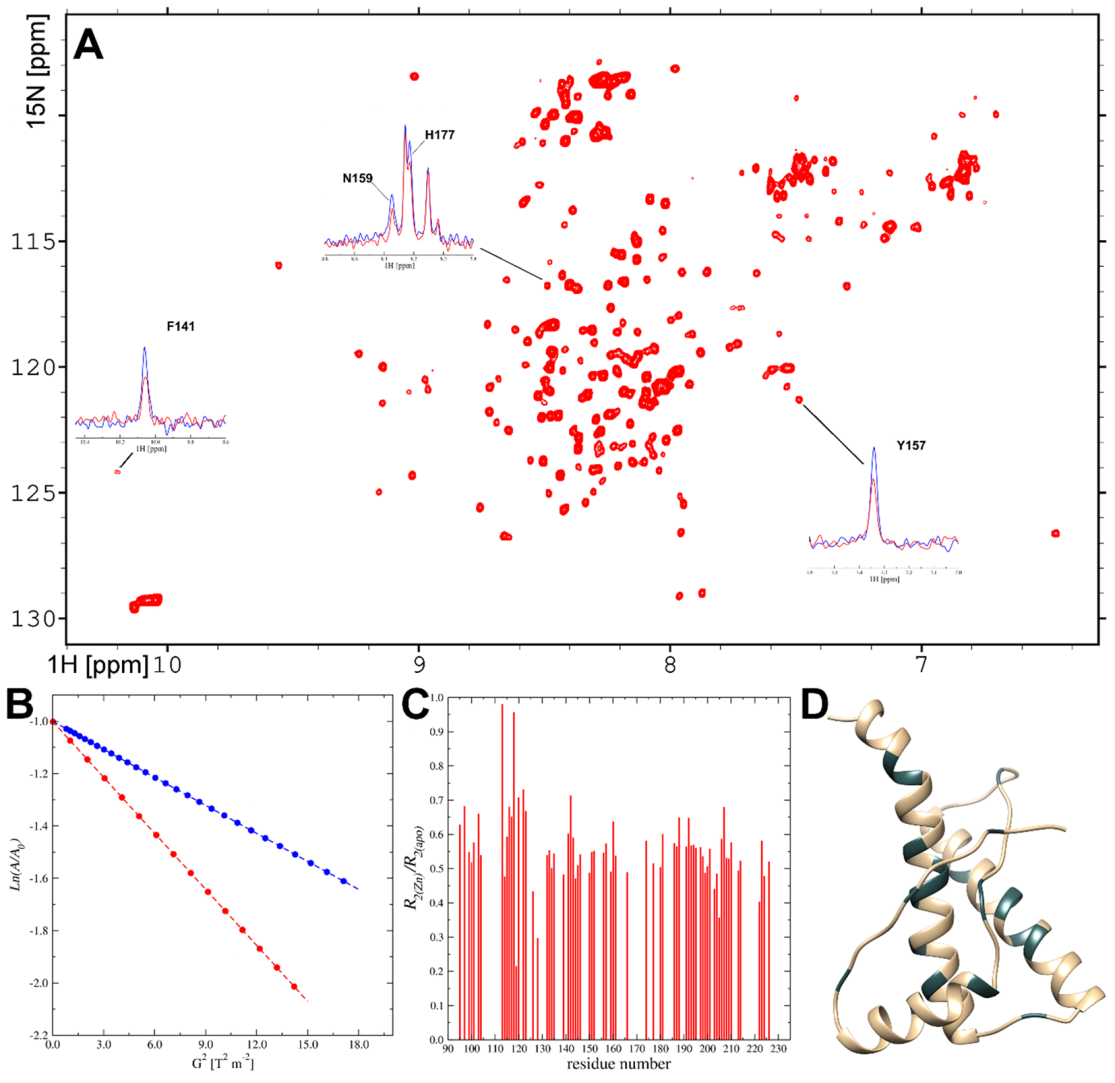
**(A)** 2D NMR ^1^H-^15^ N-HSQC spectrum of 300 µM *hu*PrP^C^ protein in 50 mM HEPES, 50 mM NaCl, pH 7.0, under saturation with Zn(II) ions. The ^1^H traces shown as insets reveal decreased peak amplitudes under Zn(II) saturation (red) compared to the spectra for the *apo* form (blue). **(B)** Relative amplitudes of the resonance peaks *versus* gradient strength, used to calculate translational diffusion coefficients for the *apo* (blue) and Zn(II)-saturated (red) forms of the PrP^C^ protein. **(C)** Relative ratios between ^15^N *R*_2_ relaxation rates obtained for the *apo* (*R*_2(apo)_) and Zn-bound (*R*_2(Zn)_) states of PrP^C^ residues. Data are presented only for residues in the C-terminal domain. **(D)** A ribbon presentation of the C-terminal fragment of the PrP^C^ protein. Residues exhibiting decreased *R*_2_ relaxation rates under saturation with Zn(II) ions are shown in dark.

To investigate the structural alteration of PrP^C^ under Zn(II) saturation, high-resolution 2D heteronuclear ^1^H-^15^ N HSQC NMR solution spectra of the protein were acquired in 50 mM deuterated HEPES buffer, 50 mM NaCl, pH 7.0, before and after addition of two molar equivalents of ZnCl_2_ (Fig. 4A). Comparison of the spectra collected without and with Zn(II) ions revealed no differences in peak positions. Reduced cross-peak intensities for certain amino acids, however, indicate residue-specific binding interactions with Zn(II) ions (Fig. 4A). As Zn(II) ions are not paramagnetic, this loss of signal intensity is likely caused by chemical exchange on an intermediate NMR time scale. Substantial intensity changes were observed for cross-peaks corresponding to amino acids in the folded domain of the PrP^C^ 3D structure—in particular in the C-terminal region with α-helices 2 and 3. In α-helix 2, decreased cross-peak amplitudes were detected for residues in the last two turns of the helix, e.g. Thr183, His187, and Thr188 (Fig. 4A).

A more compact structure of the PrP^C^ protein in presence of Zn(II) ions was demonstrated by measurements of the translational diffusion coefficient (*D*_tr_) by PGSE-NMR experiments. Addition of ZnCl_2_ resulted in higher translational mobility of the protein compared to the *apo* form, with *D*_tr_ values increasing from 1.09 ± 0.02 10^–10^ m^2^/s up to 1.23 ± 0.03 10^–10^ m^2^/s (Fig. 4B).

To further explore molecular dynamic processes in the PrP^C^ sample, the ^15^N spin–spin relaxation rates (*R*_2_) were measured. The data acquired for the Zn-bound state were compared with previously collected *R*_2_ values obtained for the *apo* form of PrP^C^ at the same conditions. For the 71 amide nitrogens assigned in the C-terminal domain, this comparison revealed decreased ^15^N *R*_2_ relaxation rates for the majority of the ^15^N backbone resonances under Zn(II) saturation (Fig. 4C). Strong effects of Zn(II)-binding were seen in a number of C-terminal residues. For example, significantly decreased *R*_2_ values for Gly126 and Tyr128 suggest changes towards more mobility for the short *β*–sheet fragment ^128^YMLG^131^. In addition, the significantly decreased *R*_2_ values for the Cys214, Thr216, Ser222, and Gln223 residues in the third α-helix suggest increased local dynamics also in this region upon Zn(II) binding (Fig. 4C).

The changes in molecular dynamic processes, observed under saturation by Zn(II), are highlighted in the 3D structure of the C-terminal domain of the protein (Fig. 4D). Together with an overall lower structural stability deduced from the ^15^N *R*_2_ data for Zn(II)-bound PrP^C^, we conclude that there are pronounced changes in the dynamics of the C-terminal region consisting of α–helices 2 and 3 joined with a β-strand motif (Fig. 4D). Our data suggest that even though local dynamics increase in the C-terminal domain by Zn(II) binding, the resulting effect is to promote interactions between the N-terminal and C-terminal domains, resulting in an overall more compact protein structure. Such an effect is in agreement with previous studies of Zn(II) binding to PrP^C^^36,38^.

### Molecular dynamics simulations show the Zn(II) binding coordination, and consequent structural and dynamic changes in the PrP^C^ C-terminal domain

**Figure 5:**
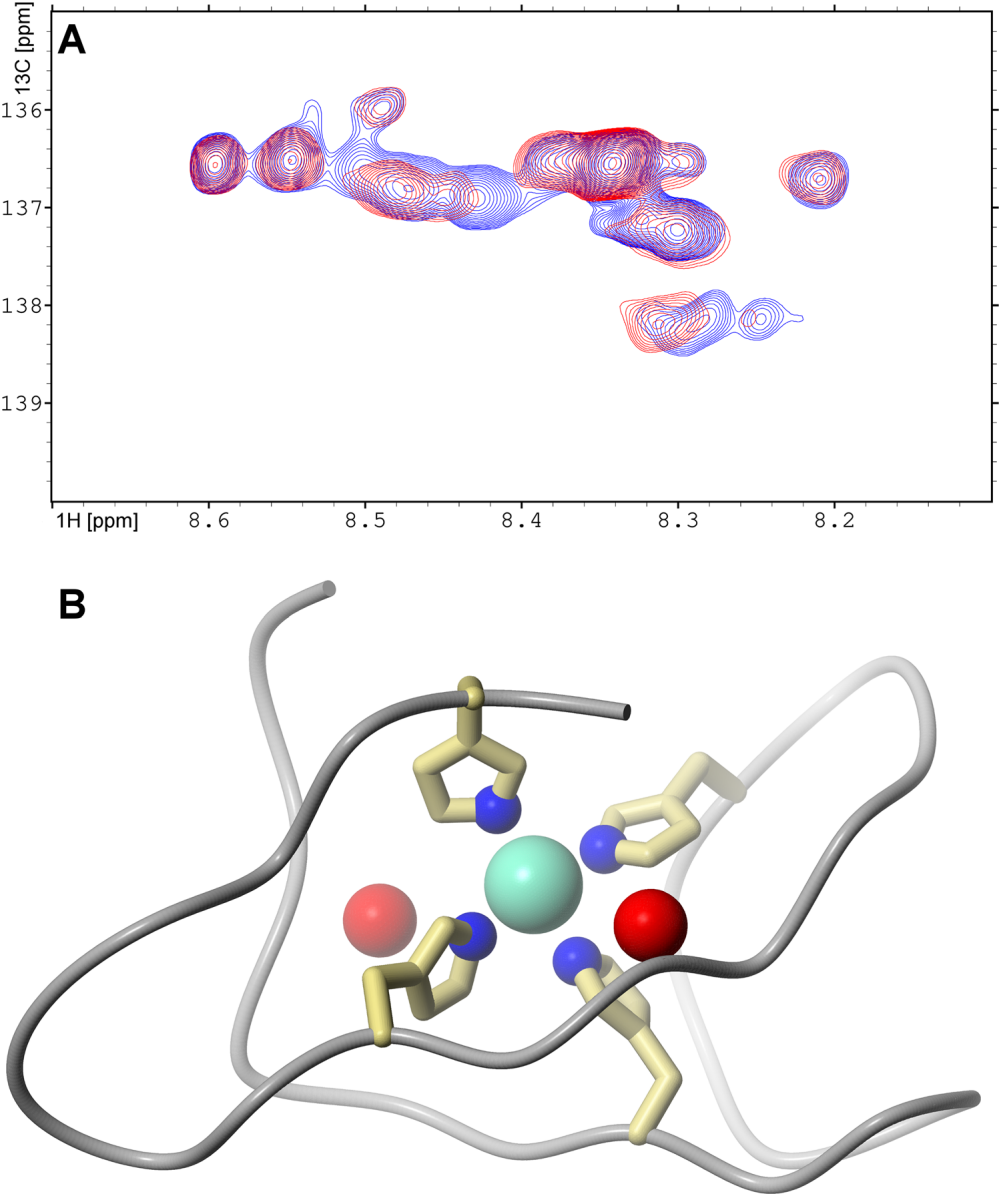
**(A)** Region of NMR cross-peaks for aromatic hydrogens, from 2D ^1^H-^13^C HSQC spectra acquired for ^13^C,^15^N-labeled *hu*PrP^C^(23–231) protein in *apo* (blue) and Zn-saturated (red) form; **(B)** 3D model of the *hu*PrP^C^ Zn(II)-binding motif, obtained from MD simulations. The histidine nitrogen atoms coordinating the Zn(II) ion are shown in blue. The two additional carbonyl oxygens (Gly71 and Trp89) present in the second coordination shell are shown in red.

As reported previously, the Zn(II) ion can be coordinated in PrP^C^(23–231) by four imidazole ring nitrogens from histidine His61, His69, His77, and His85 in the octarepeat fragment^36^. Our NMR data showed that addition of Zn(II) to PrP^C^ at pH 7.0 shifted only ^13^Cε1-^1^Hε1 resonances (Fig. S1). Despite lacking residue-specific assignments for the ^13^C and ^1^H histidine side-chain resonances, there are clear chemical shifts in four out of nine histidine ^13^Cε1 signals (Fig. 5A). We speculate that these shifts are caused by Zn(II)-binding to the four histidine residues in the octarepeat region. Thus, molecular dynamic simulations were used to create the 3D structure of the Zn(II)-binding motif shown in Fig. 5. Due to the spherical geometry of Zn(II) coordination, it is difficult to predict the exact geometry of the Zn(II)-binding motif. Nevertheless, in the second coordination sphere we note the existence of two oxygens from the carbonyl groups of Gly71 and Trp89, which may compensate for the lack of negative charges in the histidine imidazole rings^40^.

**Figure 6:**
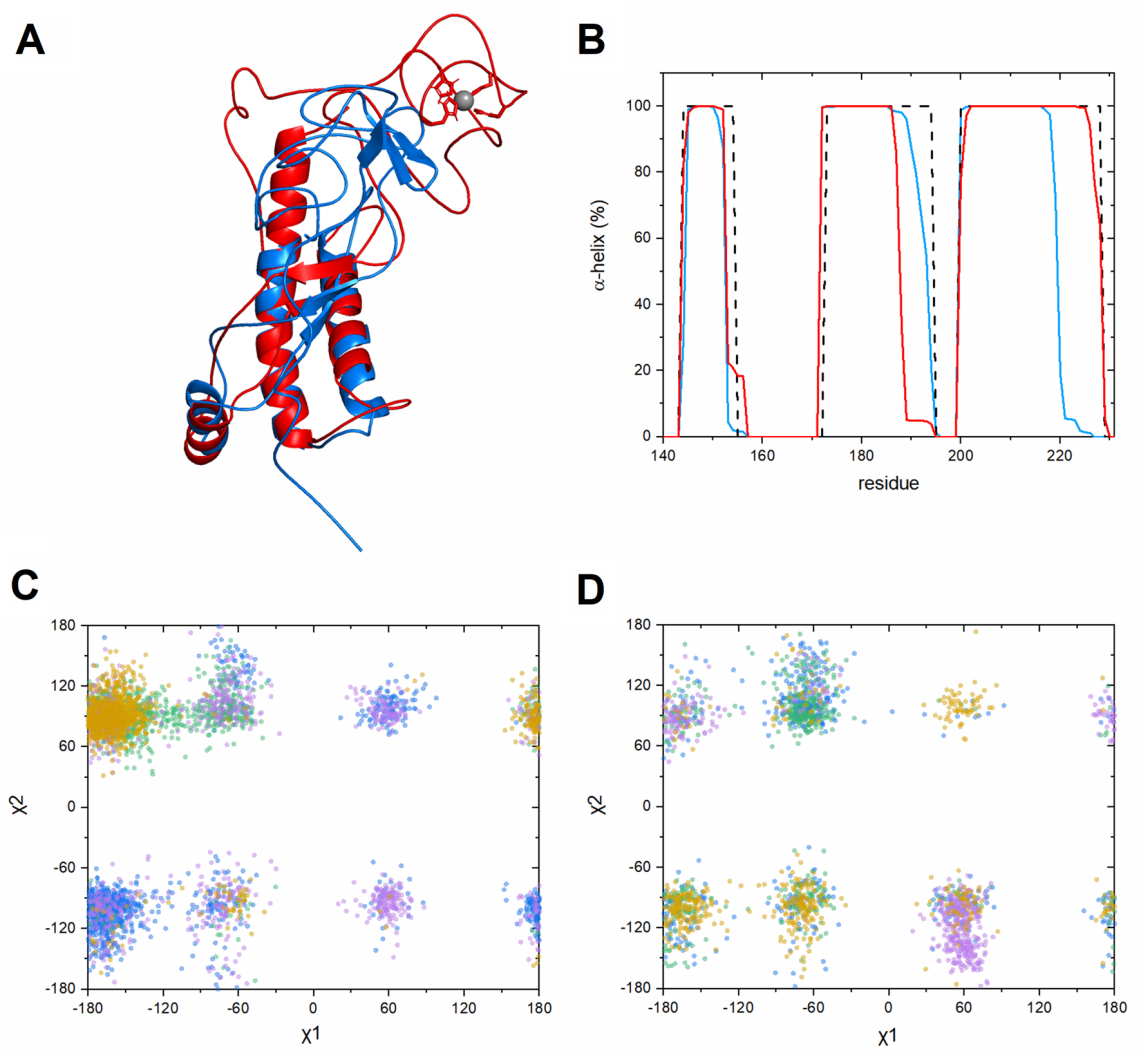
**(A)** Overlaid models of *apo*-PrP^C^ (blue) and Zn(II)-bound PrP^C^ (red), obtained at the end of the molecular dynamics simulations. The bound Zn(II) ion (gray) is coordinated by four histidine side chains from the PrP^C^ octarepeat region. **(B)** The changes in α-helicity of the PrP^C^ C-terminal domain during the whole time of the simulations for *apo*-PrPC (blue) and Zn(II)-bound PrP^C^ (red), compared to the 1QLX^13^ NMR model of PrP^C^ (dashed black line). **(C,D)** Torsion angles χ2 versus χ1, characterizing the side chain conformations of tryptophan residues from the N-terminal domain of PrP^C^ in *apo* form **(C)** and with a bound Zn(II) ion **(D)**. Trp65 is blue, Trp73 is green, Trp81 is violet, and Trp89 is yellow.

Molecular dynamics simulations were performed also to investigate changes in the C-terminal α-helices upon Zn(II) binding. In the first step, a 195 ns trajectory for *apo*-PrP^C^ with an extended N-terminal domain was performed. After ~ 25 ns of simulation the RMSD values converged (Fig. S2), and the N-terminal domain formed a compact structure with three antiparallel β-sheets (Pro51-Gly53, Gln67-His69, Gly72-Gly74) located around the C-terminal region of α-helix 3, which became partially unfolded (Fig. 6A, model marked in blue).

In the second step we generated a 100 ns trajectory for *holo*-PrP^C^ bound to a Zn(II) ion. Because our NMR experiments suggest an involvement of histidine Nε2 atoms in the Zn(II) coordination (Fig. S1), our initial model involved Zn(II) coordinated by the four deprotonated (i.e., neutral) Nε2 atoms from the His61, His69, His77, and His85 residues, located in the octarepeat region. The distances between the histidine ε2 nitrogen atoms and the Zn(II) ion were stable over the simulation time, with average values around ~ 2.15 Å (Fig. S3) and fluctuations in the range from 1.96 to 2.49 Å.

The simulation for *holo*-PrP^C^ also converged after ~ 25 ns (Fig. S2). During the simulation time the N-terminal domain moved closer to the C-terminal domain, in a similar way as for the *apo* form, although the resulting structure was less compact (Fig. 6A, model marked in red). The Zn(II)-bound octarepeat region moved to the C-terminus of α-helix 2 and unfolded this region (Fig. 6B).

**Figure 7:**
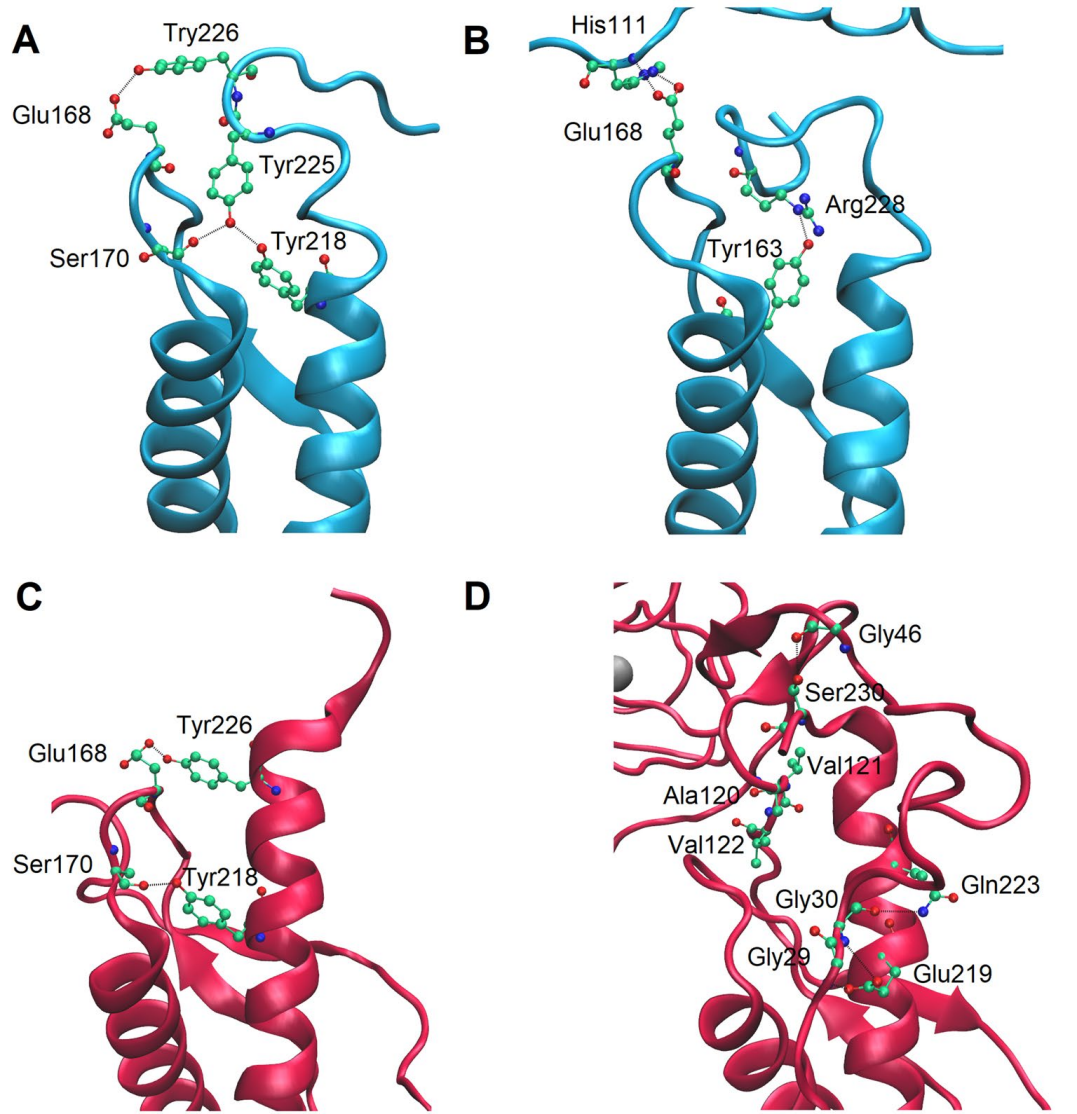
Different behavior of α-helix 3 in *apo* (blue) and *holo* (red) PrP^C^. The C-terminal region of α-helix 3 is stabilized by the β2-α2 loop **(A,C)**. In the *apo* form formation of a His111-Glu168 hydrogen bond results in rotation of Met166, which then affects the hydrogen bond formation between Arg228 and Tyr163, resulting in partial α-helix 3 unfolding **(B)**. In the *holo* form the hydrophobic amino acids Ala120, Val121, Val122 separate the β2-α2 loop from α-helix 3, and the N-terminal domain stabilizes α-helix 3 **(D)**.

Surprisingly, the main changes in PrP^C^ after Zn(II) binding involved stabilization of α-helix 3 (Fig. 6B). We therefore used MD simulations to investigate possible mechanisms for this stabilization. In *apo* form the C-terminal region of α-helix 3, involving Ser222-Arg228, becomes unfolded during a 195 ns simulation (Fig. 6B). This process may be mediated by Met166 from the β2-α2 loop. In the initial steps of the *apo*-PrP^C^ simulation, the C-terminal fragment of α-helix 3 forms hydrogen bonds with the β2-α2 loop, stabilizing α-helix 3 (Fig. 7A, Fig. S4). As the N-terminal domain can move freely, the Nδ1 atom of His111 forms a hydrogen bond with the Oε1 atom of Glu168, and disrupts the hydrogen bond between Oε2 of Glu168 and OH of Tyr226 (Fig. S5). The newly formed hydrogen bond allows Met166 to rotate (Fig. S4, Fig. S5), and then form a hydrogen bond between Sδ  of Met166 and NH2 of Arg228 (Fig. S5). In the final step, Arg228 forms a hydrogen bond involving OH of Tyr163 (Fig. 7B, S6), resulting in unfolding of the C-terminal part of α-helix 3. In difference from the *apo*-PrP^C^ simulation, such unfolding of α-helix 3 was not observed in the simulation of the Zn(II)-PrP^C^ complex. In the *holo* form, α-helix 3 is initially stabilized by the β2-α2 loop in a similar manner as initially in the *apo* form (Fig. 7C, Fig. S7). In the next step, however, the three hydrophobic residues Ala120, Val121, and Val122 intercalate between the β2-α2 loop and α-helix 3, thereby separating Met166 and Arg228 (Fig. 7D, Fig. S8). Finally, α-helix 3 becomes stabilized by the N-terminal domain, where the interaction involves four possible hydrogen bonds (Fig. 7D, Fig. S9).

Our fluorescence experiments showed a blue shift for the PrP^C^ fluorescence maximum upon Zn(II) binding (Fig. 3), which suggests less exposure to the solvent and/or partial structure induction around the tryptophan residues. To investigate if such a phenomenon correlates with our MD simulations, we analyzed the χ1 and χ2 torsion angles for all tryptophan residues. The χ2- χ1 plots for the tryptophan residues located in the octarepeat region are presented in Fig. 6C,D, while χ2- χ1 plots for other tryptophan residues are presented in Figure S10. From the seven analyzed tryptophan residues two residues from the octarepeat region (i.e., Trp65 and Trp89) showed a decrease in mobility, defined as a narrower range of preferred χ2-χ1 angles, for Zn(II)-bound PrP^C^. Interestingly, two tryptophan residues not located in the octarepeat region, i.e. Trp57 and Trp99, also showed a decrease in mobility upon Zn(II) binding. This suggests that Zn(II) binding slows down PrP^C^ dynamics not only in the octarepeat region itself, but also in the regions before and after it. In addition, χ1 for Trp81 and Trp89 from the octarepeat region more often adopted an unstable gauche^-^ conformation when Zn(II) was bound to PrP^C^. Gauche^-^ conformations were also prominent for Trp31 and Trp57, which are located outside the octarepeat region, when Zn(II) was present. This observation suggests that Zn(II) binding can act as a driving force for PrP^C^ to access a different conformational pool, that rarely is available for *apo*-PrP^C^.

### Characterization of the PrP^C^-Zn(II) complex using small angle X-ray scattering

**Figure 8:**
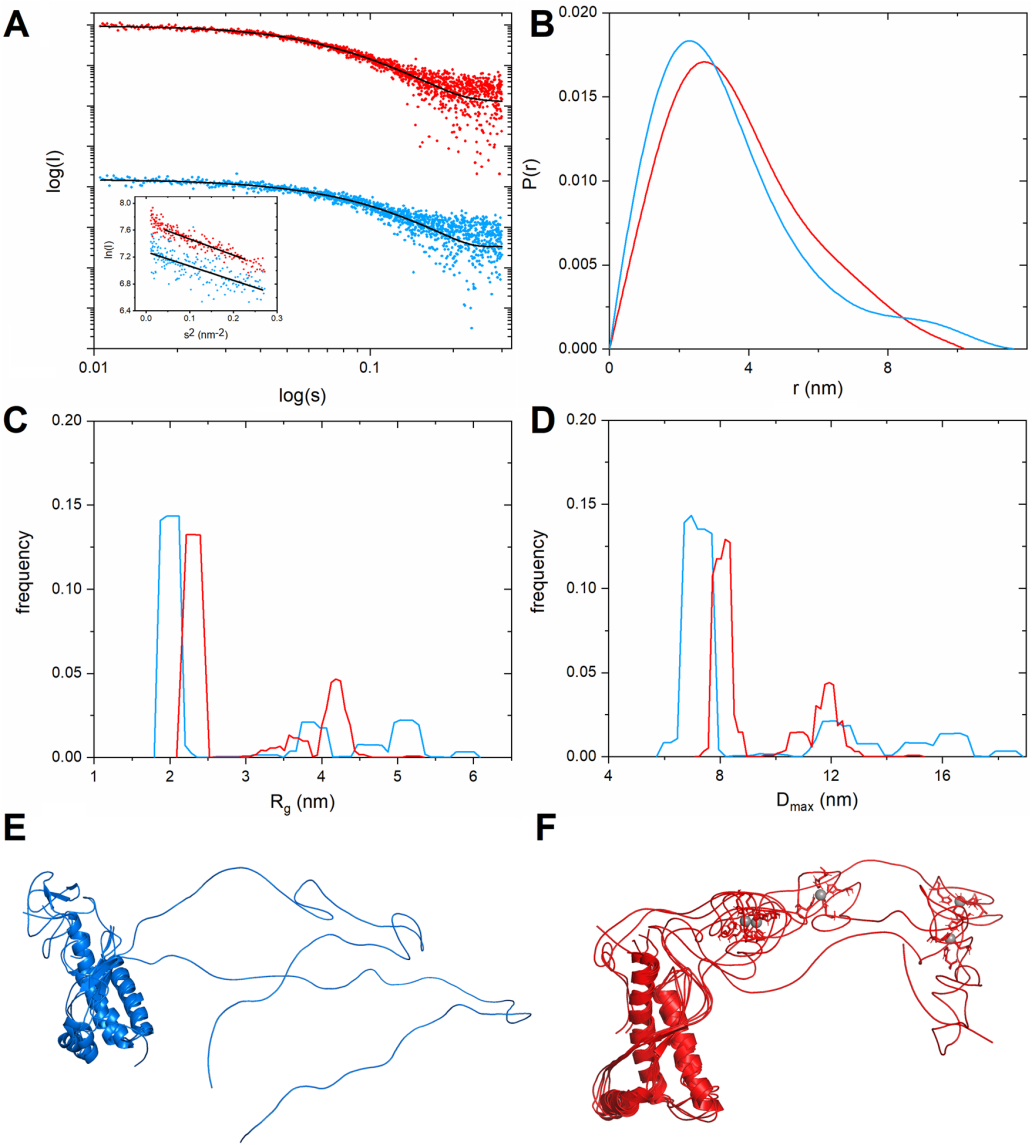
SAXS studies of PrP^C^ in *apo-* (blue) and Zn(II)-bound (red) forms. **(A)** Experimental SAXS data fitted with GAJOE (black line χ^2^ = 1.03 and 0.95, respectively). SAXS curves were displaced along the vertical axis for clarity. Left bottom inlet shows linear fits (black lines) in the Guinier regime. **(B)** P(r) functions for the two protein forms. **(C)** R_g_ distribution for the two protein forms. **(D)** D_max_ distribution for the two protein forms. **(E,F)** Pool of conformers representing *apo*-PrP^C^ **(E)** and Zn(II)-bound PrP^C^ **(F)**.

Earlier results of Spevacek et al.^36^ suggest that Zn(II) binding to the PrP^C^ protein induces a tertiary fold between the N-terminal and C-terminal domains. To investigate this hypothesis we performed small angle X-ray scattering (SAXS) experiments. In the initial SAXS analysis, we investigated scattering parameters that can be easily and directly obtained from the SAXS curve without any external structural models, namely the radius of gyration (R_g_) and the pair distance distribution function P(r). The latter is a probability function for finding two particles at a given distance, and it allows us to easily calculate the maximum diameter of a protein. The Guinier plot (Fig. 8A, inset) for *apo*-PrP^C^ and Zn(II)-bound PrP^C^ was linear (s·R_g_ < 1.3), indicating a monodisperse sample. This observation allowed us to calculate the radius of gyration (R_g_) from the Guinier approximation. Our calculated R_g_ values for the *apo*-PrP^C^ (2.51 ± 0.11 nm) and Zn(II)-bound PrP^C^ (2.66 ± 0.07 nm) were the same within experimental error. Despite no clear differences in the R_g_ values, we proceeded with calculation of the P(r) function. This function, and also the determined maximum particle diameter (D_max_), differed significantly between *apo*- and Zn(II)-bound PrP^C^. The D_max_ values were ~ 11.6 nm for *apo*-PrP^C^ and ~ 10.2 nm for Zn(II)-bound PrP^C^, suggesting a reduction in the maximum PrP^C^ diameter of ~ 1.4 nm (Fig. 8B).

At the next stage, the PrP^C^ conformation was studied by the ensemble optimization method (EOM)^41^, which is commonly used for conformational analysis of intrinsically disordered proteins or proteins with intrinsically disordered domains^42^. In this approach, the sample is treated as a polydisperse mixture of different conformers, and the experimental SAXS curve is fitted as a sum of weighted calculated scattering intensities from all conformers. As initial conformers, we used the *apo* and Zn(II)-bound PrP^C^ models generated in our molecular dynamics simulations. Thus, the SAXS curve can be represented as a population of molecules with different R_g_ or D_max_ values. In the R_g_ distribution, *apo*-PrP^C^ occupied three major and distinct conformations (Fig. 8C, blue line): compact (R_g_ ~ 2.0 nm, ~ 71%), intermediate (R_g_ ~ 4.0 nm, ~ 14%), and extended (R_g_ ~ 5.2 nm, ~ 14%). Addition of Zn(II) (Fig. 8C, red line) resulted in the appearance of a predominant conformation with R_g_ ~ 2.3 nm (~ 62%), reduction of the extended conformations to R_g_ ~ 4.2 nm (cumulatively 24%) and a reduction of the intermediate conformations to R_g_ ~ 3.4 (~ 12%). Similar features were observed in the D_max_ distribution: *apo*-PrP^C^ coexisted in three main conformations (Fig. 8D, blue line, Fig. 8E) characterized by different D_max_ values: compact (D_max_ ~ 7.5 nm, ~ 71%), intermediate (D_max_ ~ 12 nm, ~ 14%), and extended (D_max_ ~ 16 nm, ~ 14%). For PrP^C^ bound to a Zn(II) ion, D_max_ of the extended PrP^C^ conformers was shifted to ~ 12 nm (~ 24%) and ~ 11 nm (12%), with a reduction in the amount of the compact conformations to ~ 62% and an increase in D_max_ to ~ 8.1 nm (Fig. 8D, red line, Fig. 8F). Overall, the SAXS results clearly show that binding of Zn(II) ions induces a more compact fold of the PrP^C^ protein.

## Discussion

Earlier studies performed on murine PrP^C^ have indicated that the N-terminal domain becomes more ordered and interacts with the C-terminal domain upon addition of Zn(II) ions^36^. To investigate such possible effects in human PrP^C^, and if they might correspond to changes in the secondary structure of the protein, circular dichroism spectroscopy experiments were conducted. Estimation of the secondary structure from the CD spectrum of the *apo*-PrP^C^ by the BeStSel software^43^ showed an α-helical content similar to that reported for the PrP^C^ NMR structure (PDB ID: 1QLX^12^) (Table S1). The observed small discrepancy between the amounts of other structural motifs in *apo*-PrP^C^, observed by CD spectroscopy and reported in the 1QLX NMR model, are probably caused by multiple structural states of the flexible N-terminal domain^13–15^. Addition of twenty molar equivalents of Zn(II) to PrP^C^ immediately changed the CD spectrum (Fig. 2). The observed isodichroic point at ~ 242 nm suggests a decrease in the amount of regular α-helices and increase in the amount of parallel β-sheets^43^. This phenomenon probably corresponds to structural transitions within the N-terminal domain and α-helices 2 and 3, as α-helix 1 is flanked by two β-sheets (Fig. 1) and it is also more conformationally stable^12^. The proposed structural transitions would involve distortion of α-helices 2 and 3 together with structure induction in the octarepeat region with β-like motifs forming around the Zn(II) ion, rather than formation of parallel β-sheets. Such structural transitions suggest a tertiary contact between the Zn(II)-occupied octarepeat region and the C-terminal domain, in line with previous observations for mouse PrP^C^(23–230) done by Spevacek et. al^36^. The increase in the amount of distorted α-helices probably originates from contacts between the Zn(II)-saturated octarepeat region and helices 2 and 3^36^, leading to local secondary structure instabilities. According to our CD titrations the K_d_ for PrP^C^-Zn(II) in phosphate buffer is 12.1 µM. This value is close to that reported in a recently published ITC study, i.e. 16.9 µM^38^. The K_d_ values for the PrP^C^-Zn(II) complex that we here derive with different techniques (CD and fluorescence spectroscopy) and in different buffers (NEM and phosphate buffer) are slightly different, as is to be expected, but they all are in the range of 10–30 µM (Figs. 2 and 3). Thus, we conclude that the dissociation constant for the Zn(II)-PrP^C^ complex is in the low micromolar range.

Fluorescence spectroscopy also suggests structure induction in the N-terminal domain of PrP^C^ in the presence of Zn(II) ions. The fluorescence peak with a maximum at 313 nm most likely originates from tyrosine residues, as PrP^C^ does not contain any structured tryptophan residues^12,39,44^. Most tyrosine residues (nine out of twelve) are located in the C-terminal domain. Thus, minor changes in the tyrosine fluorescence signal likely correspond to changes in the local environment of C-terminal tyrosine residues. If such changes are induced by Zn(II) binding to the N-terminal region, they would arguably constitute evidence for interactions between the N-terminal and C-terminal domains.

During the titrations with ZnCl_2_ the initial fluorescence peak at 347 nm, which corresponds to multiple exposed tryptophan residues, split into two well-resolved peaks of lower intensity and with maxima at 333 nm and 343 nm. These two peaks likely correspond to tryptophan residues buried and exposed to bound water molecules, respectively^39^. This observation, which is connected also to fluorescence quenching of some tryptophan residues, suggests structure induction in the PrP^C^ octarepeat region upon Zn(II) binding. As a comparison, crystallographic studies of Cu(II) ions bound to the HGGGW fragment of the octarepeat region showed that tryptophan residues participate in Cu(II) coordination by forming a hydrogen bond with a water molecule axially bound to Cu(II)^35^. The octarepeat region appears to bind Cu(II) and Zn(II) ions via the same histidine residues, but in different binding conformations^25,29,35^. The K_d_ values calculated from our fluorescence experiments in phosphate buffer are in good agreement with our CD results, i.e. in the 10–30 µM range, which agrees with the results of previously published ICT experiments^38^. This suggests that tryptophan residues might be indirectly involved in Zn(II) binding to PrP^C^.

Our NMR data suggest that binding of Zn(II) ions to PrP^C^ produces relatively small alterations in the 3D structure of the folded C-terminal domain, as the position of the backbone amide resonances did not show significant changes. Nevertheless, Zn(II) binding leads to increased linewidths for several backbone resonances, indicating changes in spin–spin relaxation. This was confirmed by measuring ^15^N *R*_2_ values. A comparison can be made with the Amyloid-*β* (Aβ) peptide related to Alzheimer´s disease, which is well known to bind metal ions^24,45^. In analogy with the interpretations proposed in a previous NMR study of Zn(II) binding to Aβ, binding of Zn(II) to the prion protein may give rise to folding of the peptide chain around the bound metal ion^46^, which could explain the observed signal intensity changes in the HSQC spectrum. The NMR diffusion data furthermore suggest a more compact 3D structure of PrP^C^ in the presence of Zn(II), characterized by stronger interactions and an increased number of contacts between the N- and C-terminal domains, which is in agreement with the previously reported data for mouse PrP^C^^36^.

The prion protein structures deposited in PDB do not have fully defined N-terminal domain structures, as this domain is in a dynamic equilibrium between random coil, PPII helix, and β-turn secondary structures^13–15^. Two deposited structures of human PrP^C^ proteins, one for the G127V mutation and the other for wild-type PrP (5YJ4, 5YJ5), obtained from NMR and MD studies, contain in addition to the C-terminal domain also a collection of proposed models of the N-terminal domain^47^. However, such models do not answer what happens to PrP^C^ upon metal ion binding. Our MD simulations indicate that the N-terminal domain, upon Zn(II) binding, interacts with the C-terminal domain and destabilizes α-helix 2. Similar tertiary folds were previously observed for murine PrP^C^ interacting with Cu(II), Zn(II), and Cd(II) ions^36,38,48,49^, and may therefore have similar functions in metal ion recognition.

The MD simulations suggest also that PrP^C^ upon Zn(II) binding might follow a different folding path, with altered interactions between the β2-α2 loop and α-helix 3. In our proposed model for Zn(II)-bound PrP^C^, an interaction between His111 and Glu168 caused unfolding of the α-helix 3 C-terminal part. Such an interaction between His111 and the β2-α2 loop might be a first step in PrP^C^ unfolding and fibrillization. Introduction of different amino acids, in the form of glutamines in the β2-α2 loop, has previously been shown to shorten the lag phase for mouse PrP^C^ fibrillization^50^. As histidine residues at pH 7.4 can act both as donors and acceptors for hydrogen bonding, it is possible that His111 can form hydrogen bonds with glutamines from the β2-α2 loop, disturbing the α-helix 3 and resulting in faster PrP^C^ fibrillization. On the other hand, Zn(II) binding to PrP^C^ could disrupt such interactions by burying His111 in a different protein region. Previous in vitro studies on different forms of PrP^C^ seem to support this idea, as Zn(II) inhibits PrP aggregation^51,52^.

The radius of gyration for *apo*-PrP^C^ and Zn(II)-bound PrP^C^, determined by Guinier approximation, remained unchanged within experimental error, and were furthermore similar to those reported by Thakur et al. for *apo* and Cu(II)-bound PrP^C^^48^. The decrease in D_max_ after addition of Zn(II) ions indicates that the N-terminal PrP^C^ domain adopts a more compact shape in the presence of Zn(II) ions. The distance distribution function moreover suggests a reduction in the maximum particle dimension by ~ 1 nm in the presence of Zn(II), similar to what was earlier observed for the Cu(II)-PrP^C^ complex^48^. Ensemble analysis performed by a genetic algorithm produced R_g_ and D_max_ distributions similar to those for the Cu(II)-PrP^C^ complex^48^. Our results suggest that Zn(II) binding to the PrP^C^ shows similar features to those observed for Cu(II) binding, resulting in decreased N-terminal conformational freedom and therefore partial folding of the octarepeat region around the bound metal ion.

Using crosslinking, mass spectrometry, and NMR, McDonald et al. suggested that the conformational states of Cu(II)-bound PrP^C^ are available for *apo* PrP^C^ *and *vice versa, with Cu(II) acting as a switch that shifts this equilibrium^53^. Our CD, NMR and MD results suggest that PrP^C^ upon Zn(II) binding adopts a distinct conformation, that might be unavailable for *apo* PrP^C^. This distinct conformation is a result of the metal binding by the histidine residues that during coordination of a metal ion cannot form contacts with other parts of the protein. On the other hand our SAXS analysis shows the overlap between the R_g_ and D_max_ for *apo* and Zn(II)-bound PrP^C^. This might suggest that even though major conformations of *apo* and Zn(II)-bound PrP^C^ differ, some conformations are similar, which is in line with previous studies^53^.

While Zn(II) can promote dimerization of the lipid-anchored octarepeat region^54^, we have recently shown that upon interaction with Zn(II) ions octarepeat peptides (consisting of PrP(58–93)) form fibrillar structures with features characteristic for amyloids: they form the characteristic cross-β structure and bind the thioflavin T and Congo Red dyes^55^. We proposed that the reason for fibril formation could be a lack of the previously reported tertiary contact between the metal ion-saturated octarepeat region and the C-terminal domain^36,49^. Indeed, the octarepeat region seems to have an important role in PrP^Sc^ formation. Studies of antibodies targeting the C-terminal domain suggest that the octarepeat region is required to maintain high PrP^C^ toxicity, while ligands targeting octarepeat region seem to reduce PrP^C^ toxicity^56^.

Despite many years of research, the PrP^C^ function in terms of metal ion binding is still elusive, and it is still unclear if metal imbalance is part of the pathology in prion diseases. Out of three proposed functions for PrP^C^, i.e. zinc sensor, transporter, or sequester^57–59^, the first two seem to be the most tempting. With internalization of PrP^C^ into the Golgi apparatus and endosomes upon interaction with Zn(II)^60,61^, or enhancement of zinc uptake by PrP^C^ in neurons^62^ at Zn(II) concentrations around or below 100 µM, our calculated K_d_ values for the PrP^C^-Zn(II) complex in the 10–30 µM range appear reasonable and physiologically important.

In summary, our results suggest that binding of Zn(II) ions to the PrP^C^ N-terminal domain via four His residues from the octarepeat region induces a structural transition in the C-terminal α-helices 2 and 3, promotes interaction between the N-terminal and C-terminal domains, reduces the folded protein size, and modifies the internal structural dynamics. The stabilization of α-helix 3 by Zn(II) binding to the N-terminal domain may explain why Zn(II) appears to inhibit PrP^C^ fibrillization.

## Materials and methods

### Materials

The human recombinant protein PrP^C^(23–231) was expressed using previously published protocols^63,64^. The pRSETB vector (Invitrogen, USA) was used to clone plasmid containing a fusion of human PrP^C^ protein with a thrombin cleavage site and an N-terminal HisTag. The construct was expressed in *E. coli* (BL21-DE3) together with 100 µg/mL ampicillin antibiotic and induced by isopropyl *β*-D-galactopyranoside (IPTG) at OD_600_ = 0.8. A buffer containing 100 mM Tris at pH 8, 10 mM K_2_HPO_4_, 10 mM glutathione (GSH), 6 M GuHCl, and 0.5 mM phenylmethane sulfonyl fluoride (PMSF) was used during sonication of the lysates. Next, the supernatant was loaded to an Ni–NTA column (GE Healthcare) and eluted with buffer E (100 mM Tris at pH 5.8, 10 mM K_2_HPO_4_, and 500 mM imidazole). The imidazole was removed with two-step dialysis. After thrombin cleavage the *hu*PrP^C^(23–231) was concentrated using an Amicon Ultra filter (cutoff: 3 kDa).

The ^15^N-labeled and ^13^C,^15^N-double labeled forms form of *hu*PrP^C^(23–231) were prepared by the same protocol, except that the M9 media was supplemented with 1 g of ^15^NH_4_Cl and 2 g of the ^13^C-glucose (both CIL Inc, Cambridge, UK) for one liter of *E. coli* culture. Mass spectrometry was used for quality control of the samples. Protein concentrations were determined by spectrophotometry, using the extinction coefficient ε_280_ = 57,995 M^-1^ cm^-1^^65^.

### Circular dichroism (CD) spectroscopy

The initial potassium acetate buffer was exchanged to phosphate or N-ethylmorpholine (NEM) buffer using a Amicon^®^ Ultra-0.5 centrifugal filter device (Merck) with an NMWL cutoff of 3 kDa. After the first round of concentration, 10 mM phosphate, pH 7.4 or 10 NEM, pH 7.4 buffer was added to increase the sample volume up to 500 μl. The sample was then centrifuged again, and the whole procedure was repeated three times. After buffer exchange the *hu*PrP^C^ sample was filtered using an Ultrafree^®^-MC centrifugal filter with 0.22 μm pore size.

Circular dichroism spectra were collected using a Jasco J-815 spectropolarimeter (Jasco, Tokyo, Japan). The data were collected in a step scan mode with 0.5 nm resolution, 2 nm bandwidth and digital integration time of 4 s. The spectra in a 190–260 nm and 200–260 nm range were recorded in 0.1 mm and 10 mm cells respectively. All experiments were performed in triplicate with buffer baseline correction at 25 °C. Experimental data were fitted to tight binding equation^66^ (1):

1 $$\left[ \theta \right] = \left[ \theta \right]_{0} - { }\frac{{\left[ \theta \right]_{0} - \left[ \theta \right]_{\infty } }}{{2 \cdot [PrP^{C} ]}}\left( {\left( {K_{d}^{app} + \left[ {Zn} \right] + [PrP^{C} ]} \right) - \sqrt {\left( {K_{d}^{app} + \left[ {Zn} \right] + [PrP^{C} } \right)^{2} - 4 \cdot \left[ {Zn} \right] \cdot [PrP^{C} ]} } \right)$$

where [θ]_0_ and [θ]_∞_ are the initial and saturated CD intensities, [Zn] is the Zn(II) concentration, [PrP^C^] is the PrP^C^ concentration and K_d_^app^ is the dissociation constant of Zn(II)-PrP^C^ complex. Because possible binding of Zn(II) ions to the buffer is not taken into account, the calculated dissociation constants should be considered to be apparent.

Estimation of the secondary structure from the CD spectra was performed by the BeStSel software^43^.

### Fluorescence spectroscopy

Intrinsic tryptophan fluorescence was measured on a FP-8300 spectrofluorimeter (Jasco, Tokyo, Japan). Samples were excited with a wavelength of 280 nm and spectra were collected from 300 to 450 nm with 5 nm excitation and emission bandwidth. All titrations were acquired in triplicate at 25 °C. The fluorescence data were fitted with a modified Hill equation^67^ (2):

2 $$f = {\text{f}}_{\infty } + \frac{{\left( {{\text{f}}_{0} - {\text{f}}_{\infty } } \right)}}{{1 + 10^{{n_{H} \left( {log\left[ {K_{d}^{app} } \right] - log\left[ {Zn} \right]} \right)}} }}$$

where f_0_ and f_∞_ are the initial and saturated fluorescence intensities, [Zn] is the Zn(II) concentration, K_d_^app^ is the apparent dissociation constant, n_H_ is the Hill coefficient. During the fitting procedure n_H_ was 1.58 ± 0.13.

### Nuclear magnetic resonance (NMR) spectroscopy

NMR experiments were conducted at 298 K on an Agilent DDR2 800 MHz spectrometer operated at a magnetic field of 18.8 T (^1^H resonance frequency 799.838 MHz). 2D ^1^H-^15^N-HSQC and diffusion experiments were recorded for a sample containing 300 µM uniformly ^15^N-labeled human PrP^C^(23–231) protein dissolved in 50 mM HEPES-*d*_18_ (CIL, Cambridge, UK) at pH 7.0 with 50 mM NaCl added.

Due to limited amount of the ^13^C, ^15^N-double labeled protein the aromatic ^1^H-^13^C HSQC was collected for the sample prepared in a 3 mm sample tube at a concentration of 100 µM also in 50 mM HEPES-*d*_18_, 50 mM NaCl, pH 7.0. NMR spectra were recorded before and after addition of ZnCl_2_ (in two steps of respectively 50 μM and 120 μM). The recorded spectra were referenced indirectly to DSS (sodium 2,2-dimethyl-2-silapentane-5-sulfonate) using a Ξ = 0.251449530 and 0.101329118 ratio for ^13^C and ^15^N resonances, respectively^68^. All NMR data were processed with NMRPipe^69^ and analyzed with the Sparky^70^ software.

The ^15^N spin–spin relaxation rates (*R*_2_) were determined at 18.8 T using a pulse sequence based on previously published experiments^71^ and present in the BioPack library (Agilent Inc., PaloAlto, CA, USA). Due to fast relaxation of amide groups in human PrP^C^(23–231), the ^15^N *R*_2_ values were calculated only with six delays—10, 30, 50, 70, 90, and 110 ms. The experimental errors were estimated as standard deviation from 500 Monte Carlo simulations with the Relax (version 4.0.3) software^72^.

The diffusion data were collected using the DPFGDSTE (Double Polar Field Gradient Double Stimulated Echo) pulse sequence^73^. 28 and 15 data points were acquired to extract information about the translational diffusion coefficients (*D*_*tr*_), respectively for the apo and the Zn(II)-bound forms of the *hu*PrP^C^(23–231) protein. The DOSY data was processed using either VnmrJ v4.3 (Agilent Technologies Inc., USA) or MnovaNMR (Mestrelab Research SL. Santiago de Compostela, Spain) software. The *D*_*tr*_ experimental values were calculated according to the Stejskal-Tanner equation^74^ (3):

3 $$I\left( G \right) = \left( {G\gamma_{H} \delta } \right)^{2} \left( {\Delta - \frac{1}{3}G} \right)D_{tr}$$

where γ_H_ is the ^1^H gyromagnetic ratio, δ is gradient duration (2 ms), Δ is diffusion time (150 ms), and *G* is the gradient strength.

### Molecular dynamics simulations

The initial model of full length human PrP^C^ was constructed by adding the missing N-terminal domain and three C-terminal residues to the 1QLX NMR structure ^12^. The Zn(II) ion was placed near four histidine residues from the octarepeat domain using a sculpting tool implemented in pymol. Molecular dynamics simulations were performed in GROMACS 2019.2^75^ using the GROMOS 53A6^76^ force field, which contains nonbonded parameters for Zn(II). Both *apo* and Zn(II) bound PrP^C^ models were placed in a rectangular box with periodic boundary conditions (PBC) 5.0 nm from the box wall and solvated with a single point charge (SPC)^77^ water model restrained by the SETTLE algorithm^78^ using Van der Waals radii^79^. All systems were neutralized with Cl- ions and the energy was minimized with steepest descent minimization up to 5,000 steps. Temperature and pressure were equilibrated over 100 ps with a 1 fs time step using a modified Berendsen thermostat^80^ and the Parrinello-Rahman barostat^81^, respectively using the particle mesh Ewald (PME) method^82^. For both the *apo* and the Zn(II)-bound PrP^C^ molecule, the final trajectories were generated at 300 K over 195 ns with 2 fs time step or 100 ns with 1 fs time step, respectively. All covalent bonds were constrained using the LINCS^83^ algorithm. The final trajectories were analyzed in the VMD software^84^.

### Small angle X-ray scattering

The small angle X-ray scattering (SAXS) data for human PrP^C^(23–231) protein in solution were collected at the P12 beamline, operated by EMBL Hamburg at the PETRA III storage ring (DESY, Hamburg, Germany)^85^ using synchrotron radiation with a wavelength of 1.24 nm. The range of the scattering vector was from 0.105 to 3.793 nm^-1^.

For the SAXS experiments, the initial buffer was exchanged to MOPS buffer using an Amicon Ultra-0.5 centrifugal filter device (Merck) with an NMWL cutoff of 3 kDa, as described above. During the experiments the concentration of the *hu*PrP^C^ protein was 2 mg/ml. ZnCl_2_ was added from a 50 mM stock solution to a final concentration of 88 μM, corresponding to a 1:1 Zn(II):*hu*PrP^C^(23–231) molar ratio.

The SAXS data were processed and analyzed using the PRIMUS software^86^ from the ATSAS 2.8 package^87^. The radius of gyration was obtained via the Guinier approximation for s ⋅R_g_ < 1.3. The pair distribution function, P(r), and the maximum intramolecular distance, D_max_, were calculated using the GNOM software^88^. Because the N-terminal domain of *apo*-PrP^C^ is unstructured and forms multiple conformations, the SAXS data were fitted with 3D conformations from our molecular dynamics simulations using 100 cycles of GAJOE^41^.

## Supplementary Information

Supplementary Information.
