# Supplementary material for: Zn(II) binding causes interdomain changes in the structure and flexibility of the human prion protein
Source: Sci Rep. 2021 Nov 4;11:21703. doi: 10.1038/s41598-021-00495-0 (PMC8568922; doi:10.1038/s41598-021-00495-0)
Supplement: Supplementary file 1 — Supplementary Information. [file 41598_2021_495_MOESM1_ESM.docx]

**Supplementary information**

Zn(II) binding causes interdomain changes in the structure and flexibility of the human prion protein

Maciej Gielnik^1^, Michał Taube^1^, Lilia Zhukova^2^, Igor Zhukov^2^, Sebastian K.T.S. Wärmländer^3^*, Željko Svedružić^4^, Wojciech M. Kwiatek^5^, Astrid Gräslund^3^, Maciej Kozak^1,6^*

1 Department of Macromolecular Physics, Faculty of Physics, Adam Mickiewicz University,  PL 61-614 Poznań, Poland; maciejgielnik@amu.edu.pl (M.G.); mtaube@amu.edu.pl (M.T.); mkozak@amu.edu.pl (M.K)

2 Institute of Biochemistry and Biophysics, Polish Academy of Sciences, Warszawa, Poland; lilia@ibb.waw.pl (L.Z.); igor@ibb.waw.pl (I.Z.)

3 Department of Biochemistry and Biophysics, Stockholm University, 10691 Stockholm, Sweden; seb.warmlander@gmail.com (S.K.T.S.W.); astrid@dbb.su.se (A.G.)

4 Department of Biotechnology, University of Rijeka, HR 51000, Rijeka, Croatia; zeljko.svedruzic@biotech.uniri.hr (Ž.S.)

5 Institute of Nuclear Physics Polish Academy of Sciences, Krakow, Poland; kwiatek@ifj.edu.pl (W.M.K.)

6 National Synchrotron Radiation Centre SOLARIS, Jagiellonian University, PL 30-392 Kraków, Poland

*Correspondence to: [mkozak@amu.edu.pl](mailto:mkozak@amu.edu.pl), [sebastian.warmlander@protonmail.com](mailto:sebastian.warmlander@protonmail.com)

**Table S1**. Estimation of the different secondary structure components (in %) of *apo* and Zn(II)-bound PrP^C^, based on CD spectra (Fig. 2) analyzed with the BeStSel software^42^. 1QLX corresponds to the NMR structure proposed by Zahn^13^. NRMSD = normalized root-mean-square deviation.

|  | **Helix 1** | **Helix 2** | **Antiparallel β-sheet 1** | **Antiparallel β-sheet 2** | **Antiparallel β-sheet 3** | **Parallel β-sheet** | **Turn** | **Others** | **NRMSD** |
| --- | --- | --- | --- | --- | --- | --- | --- | --- | --- |
| 1QLX | 20.9 | 5.7 | 0 | 0 | 1.9 | 0 | 2.4 | 69 |  |
| *apo*-PrPC | 20.6 | 8.33 | 0.96 | 7.1 | 5.97 | 4.24 | 9.29 | 43.51 | 0.0051 |
| PrPC :Zn(II)  1:20 | 19 | 9.57 | 1.68 | 8.41 | 5.59 | 0.89 | 10.78 | 44.07 | 0.0054 |


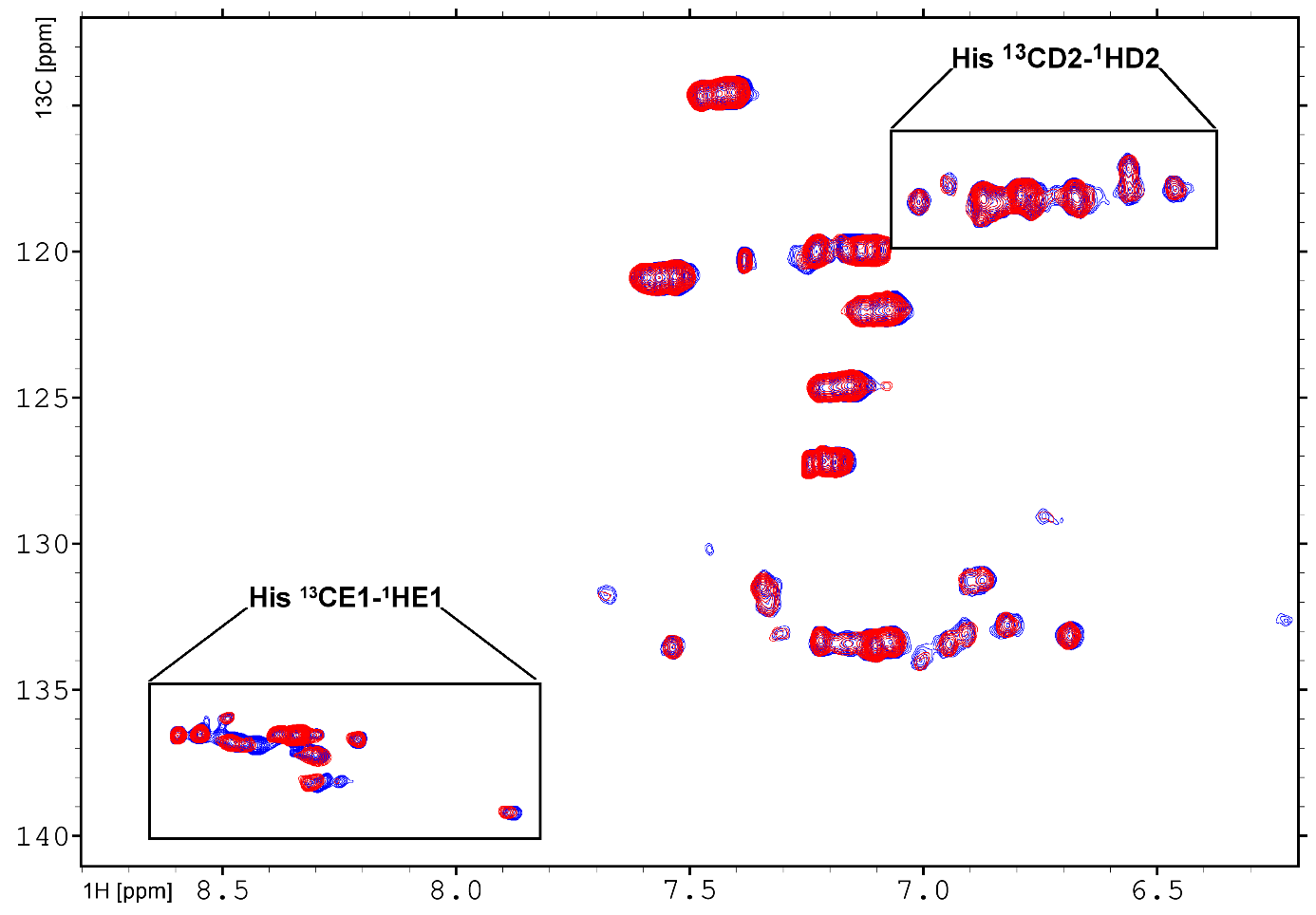


**Figure S1**. The overlay of the 2D ^1^H-^13^C HSQC spectra of the aromatic region of *apo* (blue) and Zn(II)-bound (red) PrP^C^ protein, acquired at 298 K on an Agilent DDR2 800 MHz NMR spectrometer. The regions of the ^13^Cε1-^1^Hε1 and ^13^Cδ2-^1^Hδ2 cross-peaks from histidine imidazole rings are shown as inserts.



**Figure S2**. RMSD plot for apo (blue) and holo (red) PrP^C^. Both systems converged after ~25 ns.





**Figure S3**. Computed distances between the Zn(II) ion and histidine ε2 nitrogen atoms, over 100 ns simulation time: His61 violet, His69 yellow, His77 blue, His85 red.

**Figure S4**. Possible mechanism of α-helix 3 C-terminal fragment unfolding. Initially C-terminal fragment of α-helix 3 is stabilized by hydrogen bonds between Glu168-Tyr226, Ser170-Tyr225 and Tyr225-Tyr218.


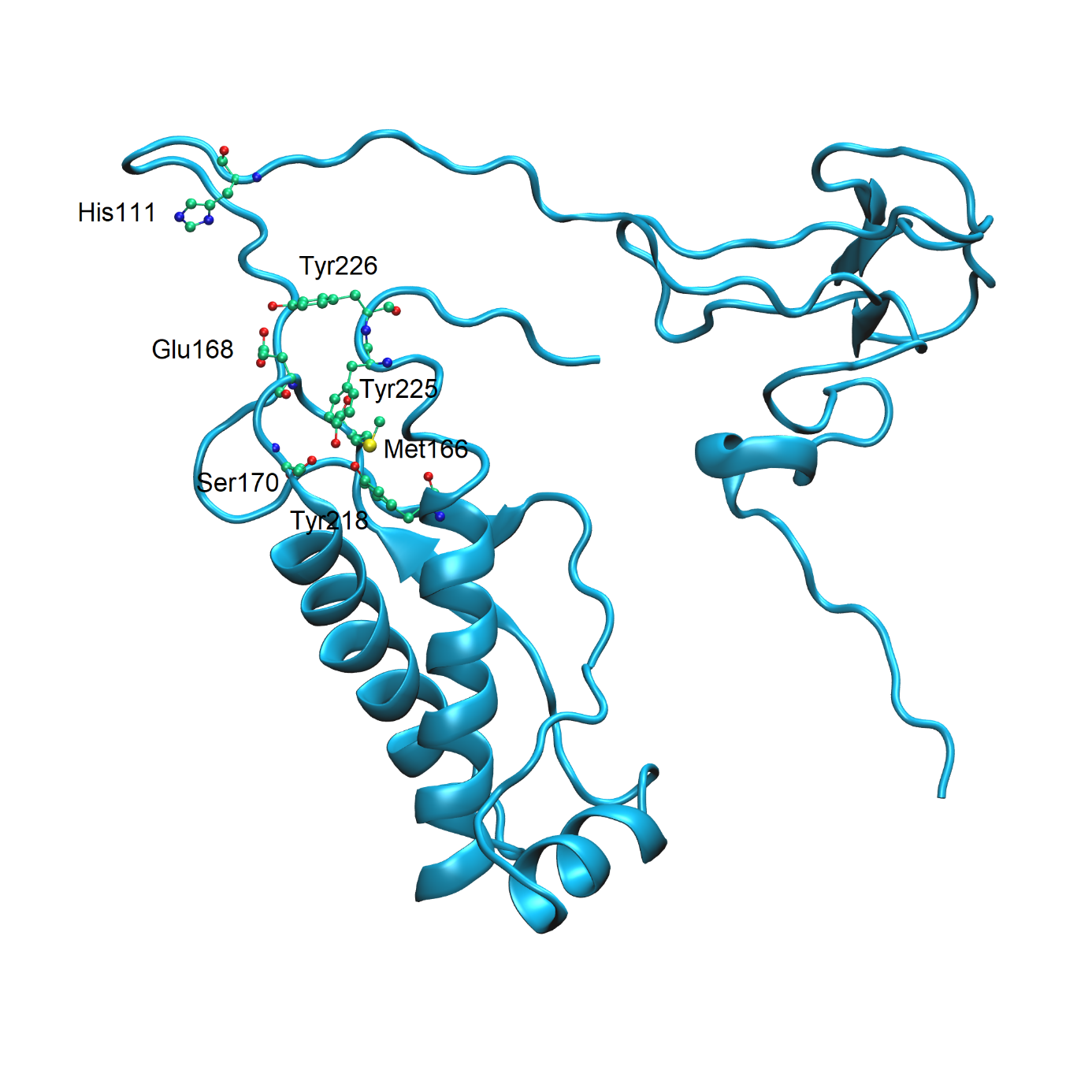


**Figure S5**. Possible mechanism of α-helix 3 C-terminal fragment unfolding. Newly formed hydrogen bond between H111-Glu168 allows Met166 to rotate and form hydrogen bond with Arg228.


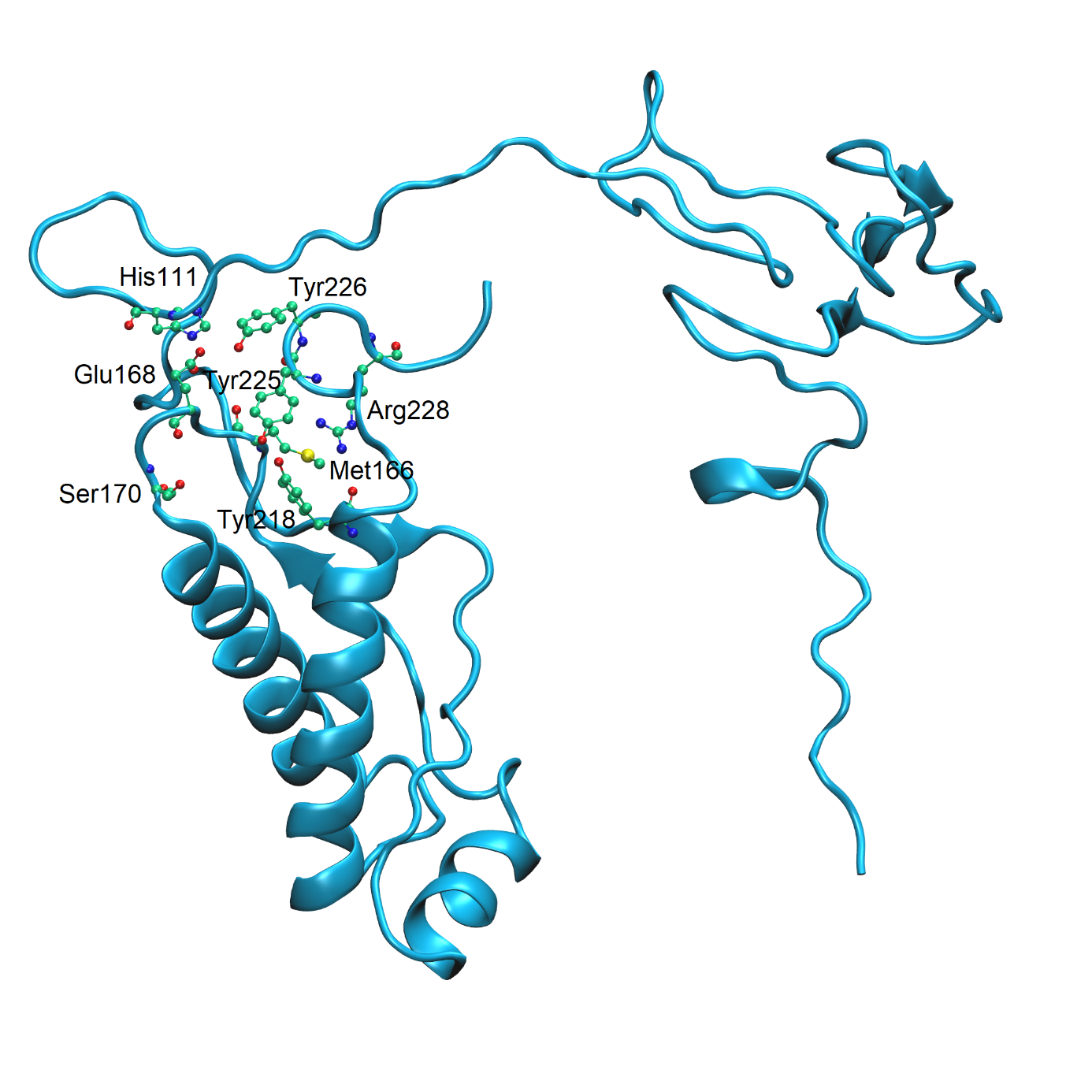


**Figure S6**. Possible mechanism of α-helix 3 C-terminal fragment unfolding. Arg228 form hydrogen bond with Tyr163 unfolding C-terminal fragment of α-helix 3.


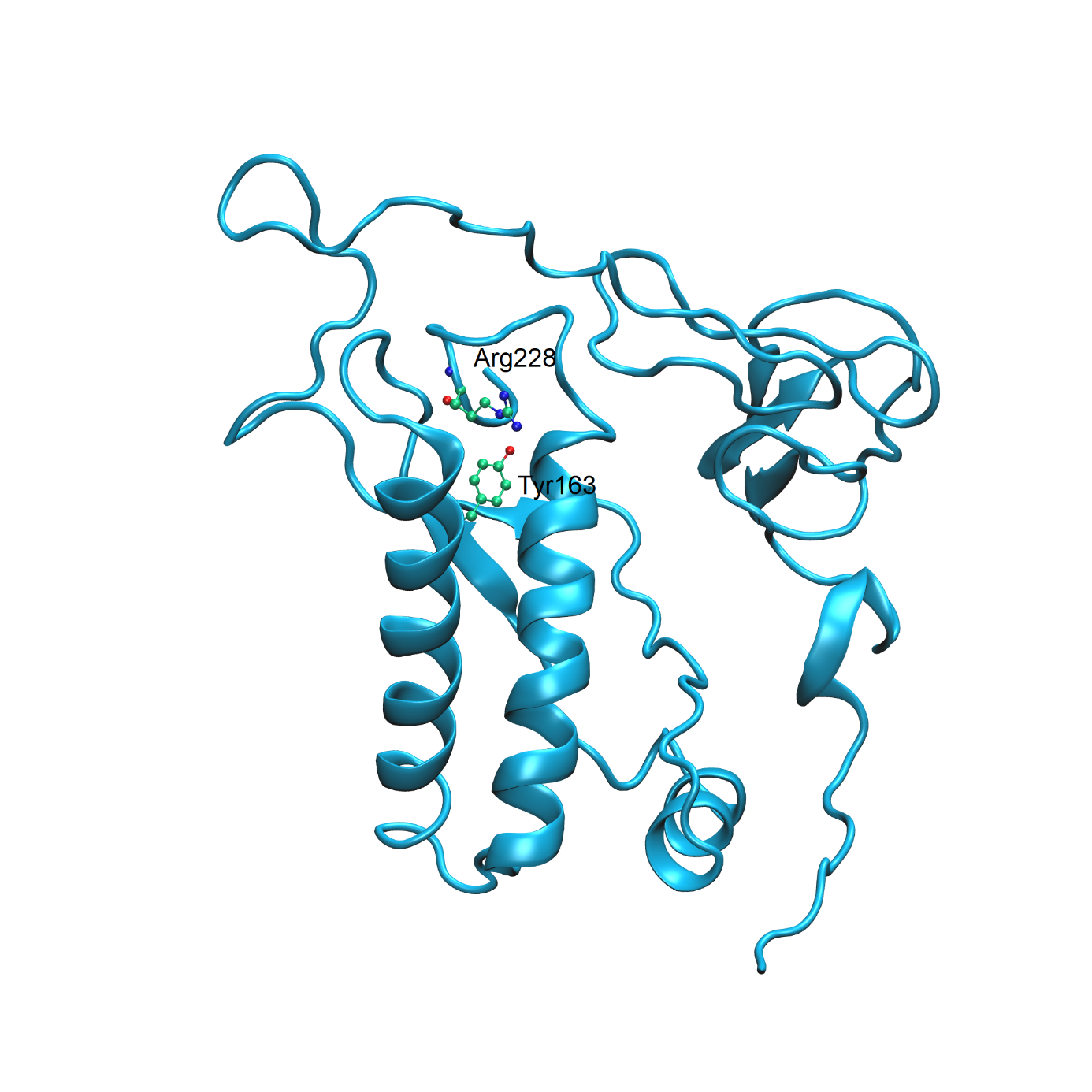


**Figure S7**. Possible mechanism of α-helix 3 C-terminal fragment stabilization in Zn(II)-PrP^C^ complex. Initially C-terminal fragment of α-helix 3 is stabilized by hydrogen bonds between Glu168-Tyr226 and Ser170-218.


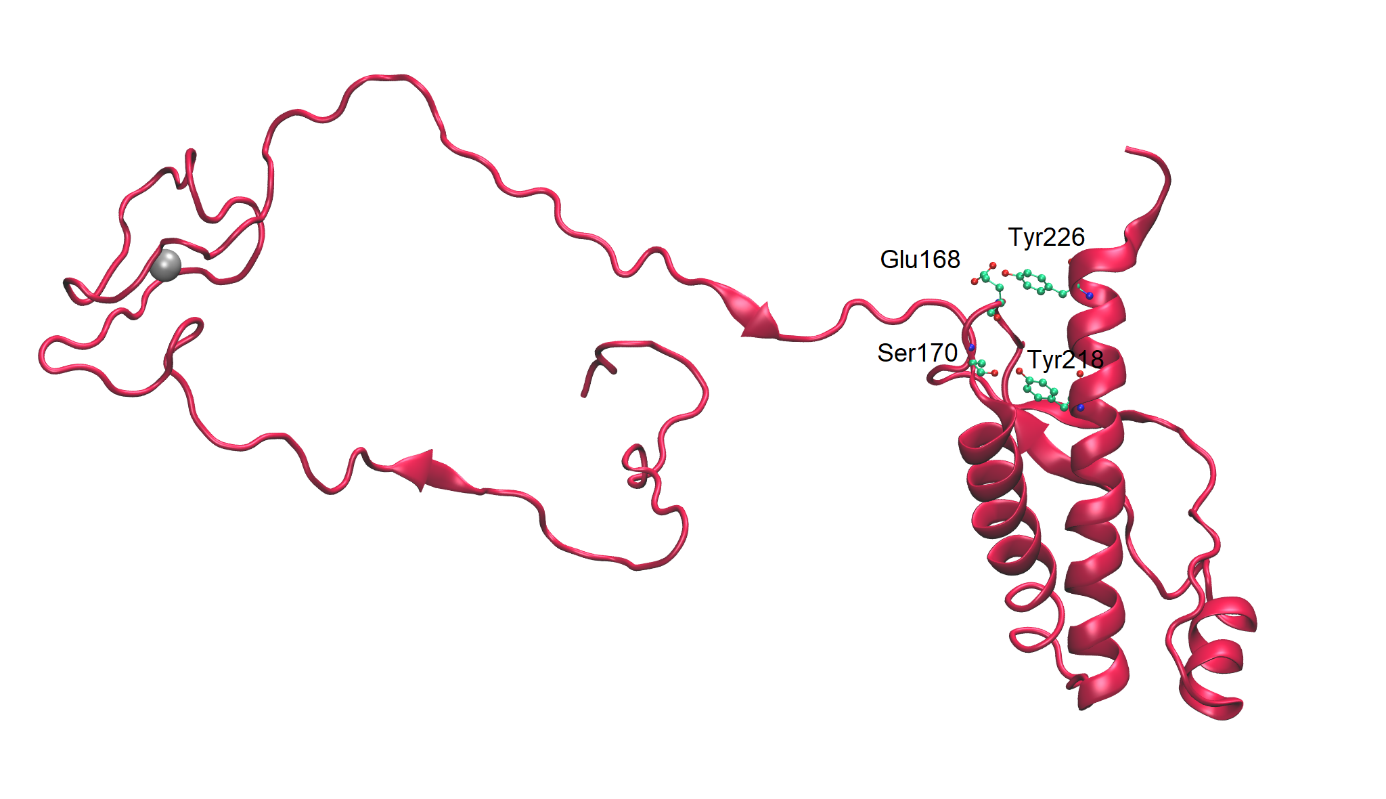


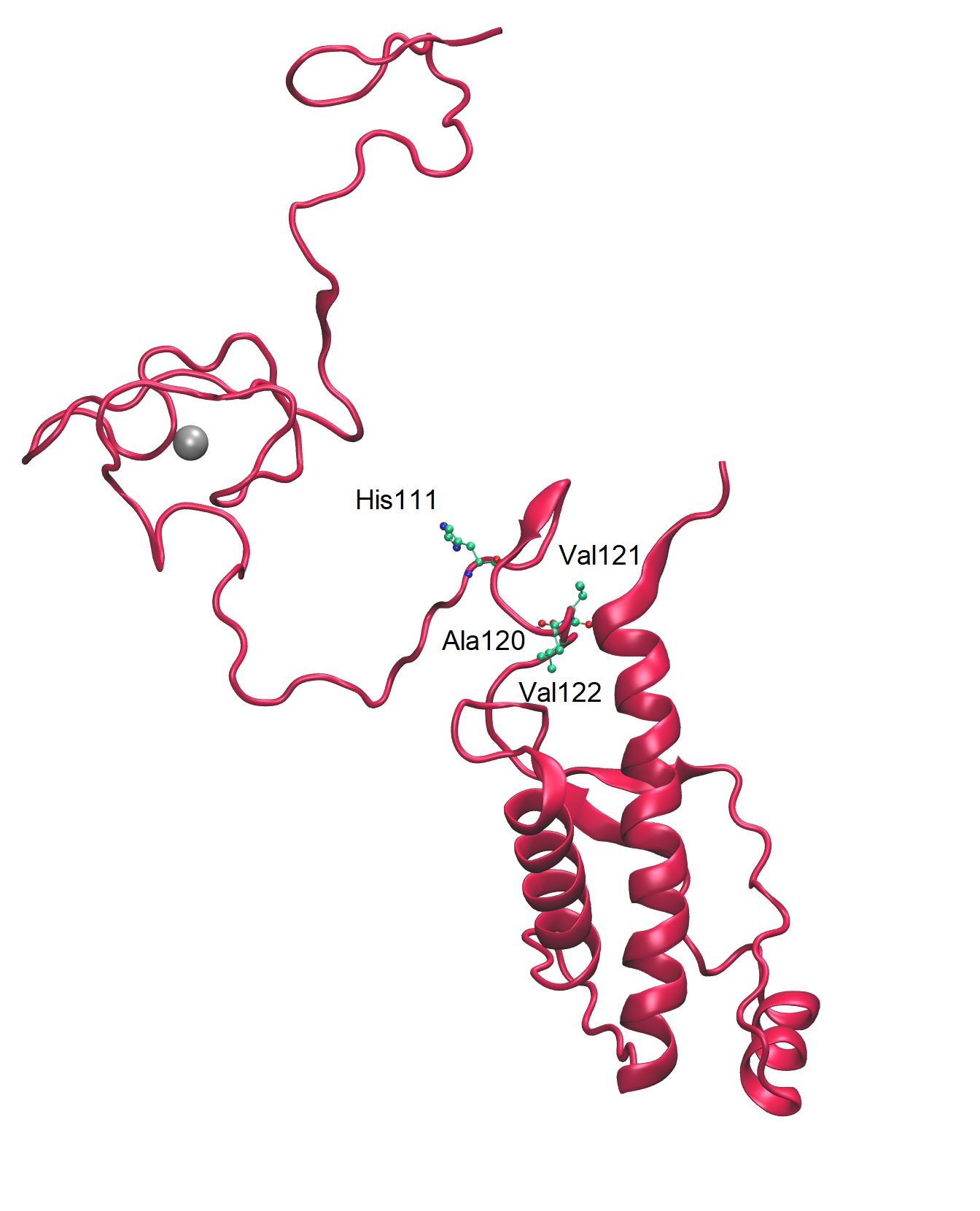
**Figure S8**. Possible mechanism of α-helix 3 C-terminal fragment stabilization in Zn(II)-PrP^C^ complex. Three hydrophobic residues Ala120, Val121, Val122 intercalate between β2-α2 loop and α-helix 3.

**Figure S9**. Possible mechanism of α-helix 3 C-terminal fragment stabilization in Zn(II)-PrP^C^ complex. N-terminal domain forms hydrogen bonds between Gly46-Ser230, Gly30-Gln223, Gly30-Glu219 and Gly29-Glu219.


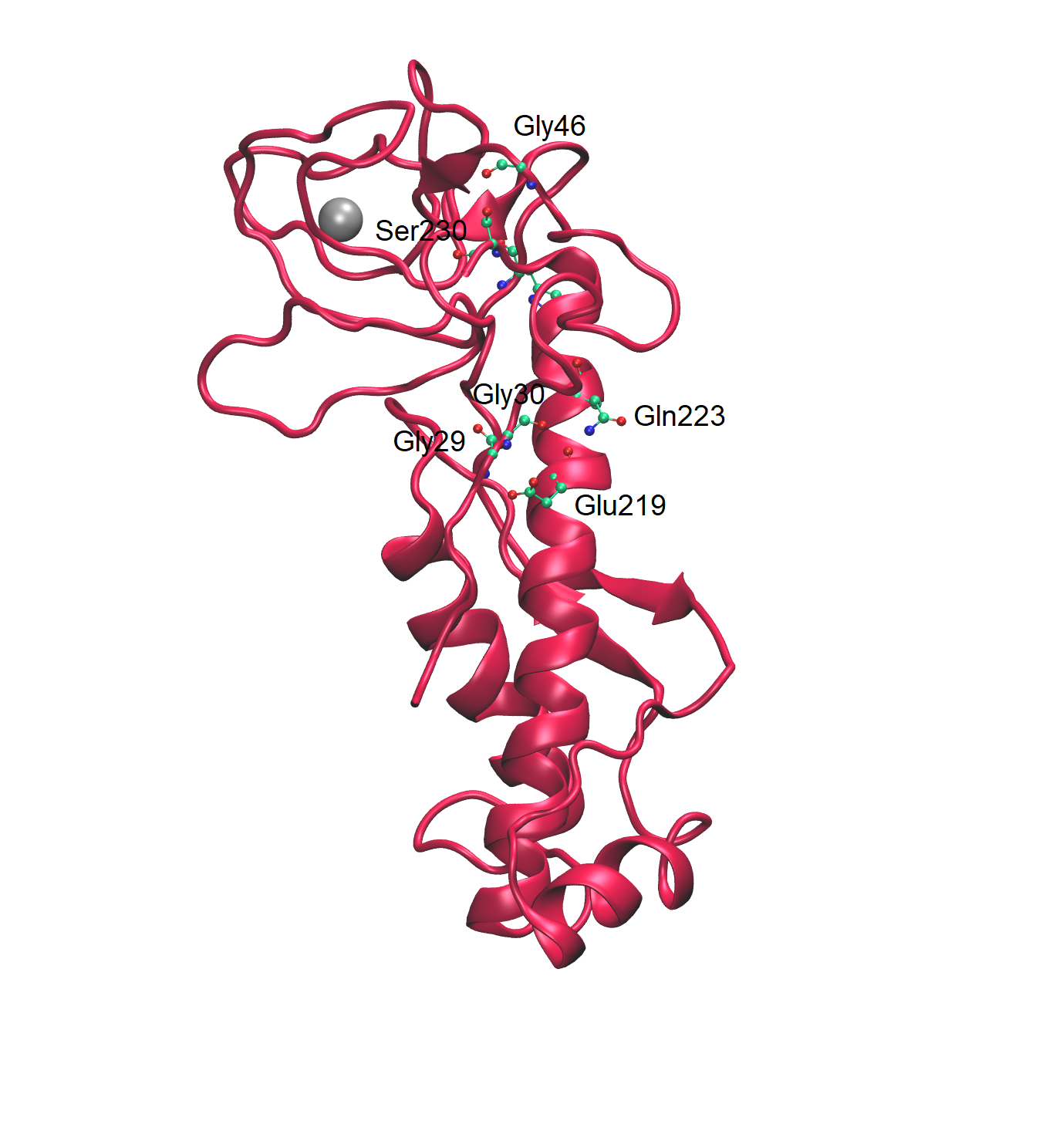


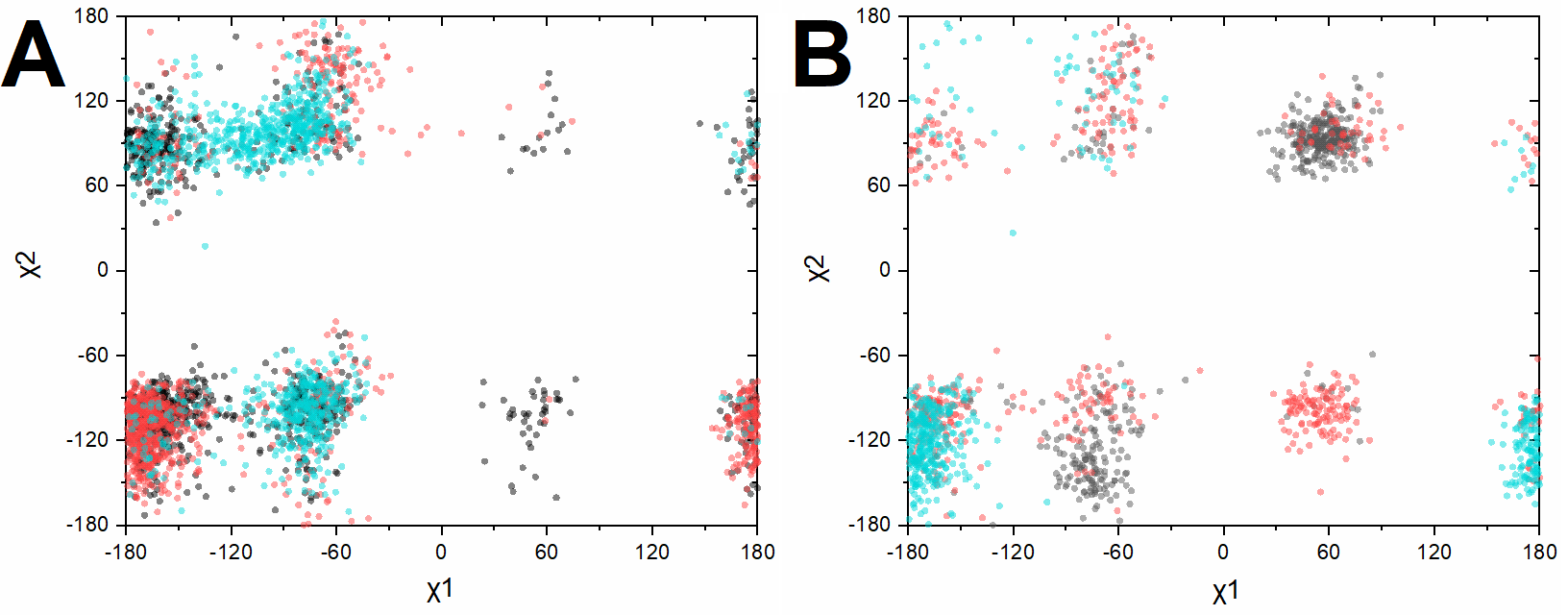


**Figure S10**. (A) Computed torsion angles χ2 versus χ1 for the side chains of tryptophan residues from the N-terminal domain of the PrP^C^ protein, in *apo* form (A) and with a bound Zn(II) ion (B). Trp31 black, Trp57 red, Trp99 blue.
